# Molecular background of the diverse metabolic profiles in leaves and inflorescences of naked catmint (*Nepeta nuda* L.)

**DOI:** 10.3389/fpls.2024.1452804

**Published:** 2024-11-28

**Authors:** Luka Petrović, Biljana Filipović, Marijana Skorić, Branislav Šiler, Tijana Banjanac, Dragana Matekalo, Jasmina Nestorović Živković, Slavica Dmitrović, Neda Aničić, Milica Milutinović, Jelena Božunović, Uroš Gašić, Danijela Mišić

**Affiliations:** Department of Plant Physiology, Institute for Biological Research “Siniša Stanković” - National Institute of the Republic of Serbia, University of Belgrade, Belgrade, Serbia

**Keywords:** metabolomics, UHPLC-ESI-QToF-MS, UHPLC/DAD/(±)HESI-MS^2^, GC/MS, phenolics, terpenes, iridoids, populations

## Abstract

*Nepeta nuda* L. shares a typical secondary chemistry with other *Nepeta* species (fam. *Lamiaceae*), characterized by the tendency to intensively produce monoterpenoid iridoids, whereas the phenylpropanoid chemistry is steered towards the production of a caffeic acid ester, rosmarinic acid. Combining complementary state-of-the-art analytical techniques, *N. nuda* metabolome was here comprehensively characterized in the quest for the organ-specific composition of phenolics and terpenoids that possess well-defined functions in plant-biotic interactions as well as therapeutic potential. *N. nuda* inflorescences showed generally higher constitutive levels of specialized metabolites, as compared to leaves, and the composition of major iridoids and phenolics in reproductive organs was found to be more conserved than in leaves across 13 populations from the Central Balkans. The results suggest that *N. nuda* plants most likely invest more in constitutive than inducible biosynthesis of functional metabolites in flowers, since they are of essential importance for both pollination and defense against herbivores and pathogens. Conversely, specialized metabolism of leaves is found to be more susceptible to reprograming in response to differential growth conditions. The defense strategy of leaves, primarily functioning in CO_2_ fixation during photosynthesis, more likely relies on the induction of metabolite levels following plant-environment interplay. Organ-specific biosynthesis of iridoids in *N. nuda* is found to be tightly regulated at the transcriptional level, and high constitutive levels of these compounds in inflorescences most likely result from the up-regulated expression of several key genes (*NnG8H*, *NnNEPS1*, *NnNEPS2*, and *NnNEPS3*) determining the metabolic flux through the pathway. The organ-specific content of rosmarinic acid and co-expression patterns of the corresponding biosynthetic genes were much less correlated, which suggests independent organ-specific transcriptional regulation of the iridoid and phenolic pathways. Knowledge gathered within the present study can assist growers to select productive genotypes and manipulate phenology of *N. nuda* towards maximizing yields and facilitating its integration into pest management systems and other applications related to human health.

## 1 Introduction

Plants produce a diverse array of specialized metabolites that have evolved specific physiological and ecological functions. These metabolites occur either as volatile or non-volatile compounds and play important roles in plant’s adaptation to different environmental conditions and defense against herbivores and microorganisms, as well as in processes such as pollination and seed dispersal (Wink, 2018). Phytochemical diversity varies across different levels of biological organization, i.e., among populations, among individuals within a population, and within an individual plant (among plant organs, during development, and across seasons) (Moore et al., 2014; Wetzel and Whitehead, 2020).

Variation in several categories of specialized metabolites is well studied at the interpopulation level of numerous species, including those belonging to the genus *Nepeta*. The interpopulation chemical polymorphism detected in *Nepeta sessilifolia*, *N. heliotropifolia*, and *N. fissa* was reported to be correlated with the elevation (Talebi et al., 2019). It has been suggested that the amounts of different types of monoterpenes decreased and the amounts of oxygenated compounds increased along the altitudinal gradient. Environmental conditions are highlighted as important factors influencing the yield and the chemical composition of essential oils of *N*. *fissa* (Talebi et al., 2017) and *N*. *heliotropifolia* (Yarmoohammadi et al., 2017). In another study, 29 accessions of *N*. *kotschyi*, *N*. *menthoides*, *N*. *crassifolia*, and *N*. *cataria* were cultivated under western Tehran (Iran) environmental conditions and evaluated for their phenolics composition (Hadi et al., 2017). Diverse composition of specialized metabolites in various plant tissues and organs is an important component of the overall plant phenotype, and changes in phytochemical profiles are one of the fastest phenotypic responses to the dynamic environmental conditions (Junker, 2016). These plant-part-specific differences may also be related to the ecological functions of the vegetative and reproductive tissues.

*Nepeta nuda* L. (subfam. Nepetoidae, fam. Lamiaceae) is an herbaceous perennial species widespread across Europe and Asia. Its individuals, characterized by numerous erect stems, oblong-lanceolate lower leaves, and ovate upper leaves can grow 50 to 100 cm tall (Aćimović et al., 2020). Flowers are organized in lax or dense spike-like verticillasters that bloom from June to August, depending on the altitude (Aćimović et al., 2020). Health benefits of *N*. *nuda* are usually associated with the accumulation of terpenoids and phenolics, two major groups of specialized metabolites of the genus *Nepeta.* Terpenoids, predominately represented by iridoid compounds, are produced in most plant tissues where they primarily act either as pollinator attractants (Schultz et al., 2004) or insect repellents (Bernier et al., 2005; Birkett et al., 2011; Sparks et al., 2018; Reichert et al., 2019; reviewed in Formisano et al., 2011 and Süntar et al., 2018), but can also function as insect pheromones (Glinwood et al., 1999; Birkett and Pickett, 2003) and are appealing to felines (Uenoyama et al., 2021 and 2022). Numerous studies on the chemical composition of *N*. *nuda* plant extracts and essential oils revealed a high level of chemodiversity, which may be ecologically driven, as it has been proven to be strongly influenced by various environmental factors, such as geographical origin, light conditions or altitude (Sharma et al., 2021; Petrović et al., 2024). Only few studies report on variations in iridoid and phenolic composition among populations of *N*. *nuda* within a specific geographical region (Narimani et al., 2017; Petrova et al., 2022; Petrović et al., 2024). In a recent study, we reported low differences in the iridoid and phenolic profiles in leaves among populations in the Central Balkans, since the majority of chemical and genetic variations were found within populations (Petrović et al., 2024). Moreover, other recent studies suggested organ-specific biosynthesis and accumulation of bioactive metabolites in *N*. *nuda* (Petrova et al., 2022; Zaharieva et al., 2023). Thus, the content of rosmarinic acid and 1,5,9-*epi*-deoxyloganic acid in *N*. *nuda* plants from Mt. Pirin (Bulgaria) was significantly higher in flowers than in leaves (Petrova et al., 2022).

The objective of this study was to comparatively investigate the patterns of metabolites’ variation in *N*. *nuda* vegetative (leaves) and reproductive (inflorescences) organs across 13 natural populations within the Central Balkans, adopting both targeted and untargeted metabolomics approaches. The determination of variation patterns in the plant metabolome in nature is crucial for predicting the persistence of populations and species in the future, understanding the species’ ecological interactions, and conserving their genetic diversity (Labarrere et al., 2019). Characterization of intraspecies chemodiversity in *N*. *nuda* within the Central Balkans can indirectly enable mapping of areas suitable for cultivation and intendedly assist farmers and other stakeholders to identify accessions with the highest crop potential and earmark them for future cultivation. *N*. *nuda* can be considered attractive medicinal crop for montane and subalpine regions (up to 2100 m a.s.l.), due to high adaptive success and production of essential oils with prominent bioactive properties (e.g., Gkinis et al., 2010; Formisano et al., 2011; Musso et al., 2017; reviewed in Sharma et al., 2021; Zaharieva et al., 2023). Considering differential but equally important ecophysiological roles of leaves and inflorescences, we intended to investigate which plant part is more susceptible to metabolic reprogramming in response to external influences and discuss the potential ecological implications arising from variations in the composition of terpenes and phenolics in these two organs of *N*. *nuda*. We further delved into the molecular background of the organ-specific differences in the biosynthesis and accumulation of iridoids and phenolics, thus providing evidence for their mutually independent transcriptional regulation of metabolic fluxes in different organs.

## 2 Materials and methods

### 2.1 Chemicals and reagents

Acetonitrile (Fisher Scientific UK, Leicestershire, UK) and formic acid (Merck, Darmstadt, Germany) were of MS grade. Ultra-pure deionized water was generated using a Water Purification System (New Human Power I Integrate, Human Corporation, Republic of Korea). Standards of 1,5,9-*epi*-deoxyloganic acid and 5,9-dehydronepetalactone were isolated from natural sources as previously described by Aničić et al. (2021). The standard of *cis,trans*-nepetalactone was a generous gift from Entomol Products LLC (San Francisco, CA, USA). Analytical standards of loganin, rosmarinic acid, caffeic acid, luteolin and apigenin were purchased from Sigma-Aldrich (Hamburg, Germany).

### 2.2 Collection of plant material

Aboveground parts of flowering *N. nuda* plants were collected in June-August 2022 from 13 populations across Serbia: 1. Mali Ljukten (43°32’35”N; 20°48’38”E); 2. Brodica (44°29’04”N; 21°50’27”E); 3. Debeli lug (44°21’45”N; 21°54’01”E); 4. Rtanj (43°44’13”N; 21°57’03”E); 5. Straža (43°50’46”N; 21°42’03”E); 6. Židilje (44°00’42”N; 21°38’45”E); 7. Vinatovača (44°04’14”N; 21°45’36”E); 8. Vlasina (42°41’39”N; 22°22’44”E); 9. Donji Krivodol (43°06’18”N; 22°55’41”E); 10. Topli Do (43°22’05”N; 22°37’58”E); 11. Janjska reka (43°25’33”N; 22°31’11”E); 12. Balta Berilovac (43°24’10”N; 22°30’43”E); 13. Gornje selo (42°11’21”N; 20°56’20”E). Plants were identified in the field by the authors, and representative specimens, assigned with the voucher numbers, have been deposited in the Herbarium of the Institute of Botany and Botanical Garden, University of Belgrade, BEOU (acronym follows Thiers, 2023). Plant material was dried in the shade at room temperature until constant mass and subsequently stored in paper bags. Samples were kept in the dark at room temperature until use. *N. nuda* aboveground parts were detached to leaves and inflorescences.

### 2.3 Preparation of *N. nuda* methanol extracts

Samples containing either leaves or inflorescences (50 mg) of *N. nuda* were ground in liquid nitrogen and extracted overnight at 4 °C with 1 mL of 96% methanol (w:v = 1:20). After vortexing for 1 min, samples were sonicated in an ultrasonic bath (RK100, Bandelin, Berlin, Germany) for 1 h at 4°C. Supernatants were separated after centrifugation at 10,000g for 10 min and subsequently filtered through 0.2 µm-pore size cellulose filters (Agilent Technologies, Santa Clara, CA, USA). Each population was represented by 3 biological replicates.

### 2.4 Characterization of metabolites in *N. nuda* leaves and inflorescences using UHPLC-ESI-QToF-MS analysis

Three randomly selected *N. nuda* samples (populations 3, 8, and 9) of both inflorescences and leaves were subjected to metabolic fingerprinting using an Agilent 1290 Infinity UHPLC system in combination with a quadrupole time-of-flight mass spectrometer (6530C QToF-MS, Agilent Technologies, Inc., Santa Clara, CA, USA). The mass detector, equipped with an electrospray ionization (ESI) source, was operated in both positive and negative ionization modes, in the mass range from 100 to 1000 *m/z*. Samples were chromatographically separated on a Zorbax C18 column (2.1 × 50 mm, particle size 1.8 µm; Agilent Technologies, Inc., Santa Clara, CA, USA) thermostated at 40°C. The gradient elution program, UHPLC and MS parameters as well as ion source settings were described by Petrović et al. (2024).

The Agilent MassHunter software was used for acquisition, control, and MS data collection from the 6530C Q-ToF-MS instrument. The R Studio platform (‘enviPick’ and ‘xcms’ R packages) was used for the evaluation and presentation of MS data (Zengin et al., 2021). The metabolites were identified based on their monoisotopic masses and MS^2^ fragmentation and confirmed using literature data (Alimpić Aradski et al., 2023; Aničić et al., 2021; Dienaitė et al., 2018; Goldansaz et al., 2019). Accurate masses of components and fragment ions were calculated using the ChemDraw software (version 12.0, CambridgeSoft, Cambridge, MA, USA). The CAS SciFinder-n database (https://scifinder-n.cas.org/) was used to search for chemical compounds by formulae and structures.

### 2.5 UHPLC/DAD/(±)HESI-MS^2^ targeted metabolic profiling of *N. nuda* leaves and inflorescences

A targeted UHPLC/DAD/(±)HESI/MS^2^ metabolomics approach was adopted to quantify individual phenolics (rosmarinic acid, caffeic acid, apigenin, and luteolin) and iridoids (*cis,trans*- and *trans,trans*-nepetalactone, 5,9-dehydronepetalactone, nepetanudoside A, and 1,5,9-*epi*-deoxyloganic acid) in *N. nuda* methanol extracts of leaves and inflorescences. Analyses were performed on a Dionex UltiMate 3000 UHPLC system coupled with a DAD detector and configured with a triple quadrupole mass spectrometer (TSQ Quantum Access Max, Thermo Fisher Scientific, Basel, Switzerland). Samples were chromatographically separated on a Hypersil gold C18 analytical column (50 × 2.1 mm, 1.9 µm particle size; Thermo Fisher Scientific, Waltham, MA, USA), using the elution gradient and the flow rate previously described by Aničić et al. (2021). The mobile phase consisted of (A) water + 0.1% formic acid and (B) acetonitrile + 0.1% formic acid. The injection volume was 10 µL. The selected reaction monitoring (SRM) mode of the instrument was used for the quantification of the targeted compounds by direct comparison with the commercial standards. Nepetanudoside A was quantified based on the calibration curve of loganin, while quantification of aucubin was performed using the calibration curve for nepetaside. Calibration curves of pure standards revealed good linearity, with *r* ^2^ values exceeding 0.99 (peak areas vs. concentration). The total amount of each phenolic and iridoid compound was evaluated by the calculation of its peak area and is expressed as µg per 100 mg leaf/inflorescence DW.

### 2.6 GC/MS non-targeted metabolomics of methanol extracts of *N. nuda* leaves and inflorescences

Profiling of volatile compounds in methanol extracts of *N. nuda* leaves and inflorescences was performed using an Agilent 8890 gas chromatography (GC) system coupled with a Mass Selective Detector (5977B GC/MSD, Agilent Technologies, Santa Clara, CA, USA) and connected to an automated sample extraction and enrichment platform (Centri^®^, Markes International Ltd., Bridgend, UK). Chromatographic separation procedure is described in detail by Petrović et al. (2024). The constituents of the methanol extracts were identified by comparison of their mass spectra and retention times with those of the respective standards as well as by comparison with the NIST05 library.

### 2.7 Statistical analyses of metabolomics data

For hierarchical cluster analysis (HCA), the input variables were scaled to the [min, max] range. HCA was performed based on the Spearman’s method of cluster agglomeration, using the Morpheus software (https://software.broadinstitute.org/morpheus). Principal component analysis (PCA) was constructed using the Past 4 software (version 4.14; Hammer and Harper, 2001). For the comparison between populations, quantitative metabolomics data were subjected to the Tukey’s *post hoc* test (p < 0.05) of one-way ANOVA. To test the statistical difference between leaves and inflorescences, *t* test was adopted.

### 2.8 Comparative metabolic profiling and gene expression analysis in leaves and inflorescences of greenhouse-grown *N. nuda*

#### 2.8.1 Plant material and metabolic profiling of iridoids and phenolics

Seeds of *N. nuda*, collected in June 2022 in the locality Debeli Lug (East Serbia) were germinated under *in vitro* conditions, as previously described (Aničić et al., 2024). Following clonal propagation of a single genotype under *in vitro* conditions, adopting previously optimized protocols for other *Nepeta* species (Aničić et al., 2024), plantlets were acclimatized in a greenhouse of the Institute for Biological Research “Siniša Stanković” - National Institute of the Republic of Serbia, University of Belgrade, Serbia. Samples of leaves and inflorescences were collected from fully-flowering plants in their third year of growth under greenhouse conditions.

Leaves and inflorescences were excised from plants and immediately frozen in liquid nitrogen (LN). Samples were mechanically grinded in LN until fine powder, weighted and aliquoted into batches of 100 mg, and subsequently stored at −80°C until use. The first group of batches containing three biological replicates, designated for metabolomics, was dried by lyophilization until constant mass, weighted, and extracted with 96% methanol (100:1 = w:v). Following vigorous vortexing for 1 min, the extraction was continued in an ultrasonic bath for 1 h, and supernatants were separated by centrifugation for 10 min at 10,000*g*. Supernatants were filtered through 0.2-mm cellulose filters (Agilent Technologies, Santa Clara, CA, USA) and stored at 4°C until use. Quantification of the targeted iridoids and phenolics in samples was performed using a UHPLC-DAD-(±)HESI/MS^2^ instrument as described in section 2.5.

#### 2.8.2 RNA extraction and qPCR assessment of the expression of biosynthetic genes

The second batch of samples was used to extract RNA from *N. nuda* leaves and inflorescences, and all the extractions were performed in three biological replicates. The RNA extraction was performed applying a modified CTAB method (Gasic et al., 2004), which was followed by the quantification using a N60 Nano-Photometer^®^ (Implen GmbH, Munich, Germany) and a Qubit 3.0 Fluorometer (Thermo Fisher Scientific, Waltham, MA, USA), while the RNA integrity was electrophoretically checked. The procedure of the DNase I (Termo Fisher Scientific, USA) treatment and the protocol for cDNA synthesis were described earlier by Aničić et al. (2024).

Gene expression analyses were performed by real-time PCR using QuantStudio™ 3 Real-Time PCR System (Thermo Fisher Scientific, Carlsbad, CA, USA). Thermocycler conditions were previously described by Aničić et al. (2018). The reactions were performed using Maxima SYBR Green/ROX Master Mix (2X) (Termo Fisher Scientific, USA), cDNA corresponding to 50 ng RNA, and 0.3 µM primers, according to the manufacturer’s recommendations. The expression levels of targeted candidates of iridoid- and phenolic-biosynthetic genes were calculated according to the 2^–ΔΔCt^ method (Livak and Schmittgen, 2001) using GAPDH as a housekeeping gene. Primer pairs were designed based on candidate genes whose sequences and accession numbers are listed in **Supplementary Table 1**.

#### 2.8.3 Statistical analysis of gene expression data

qPCR data were processed and visualized using the Past 4 software (version 4.16; Hammer and Harper, 2001). To test the statistical difference between leaves and inflorescences, *t* test was adopted. Correlation analysis, adopting the Ward’s algorithm, was performed to examine the co-expression patterns of targeted genes in leaves and inflorescences of *N. nuda*.

Linear regression analysis was performed to visualize relations between metabolomics and gene-expression data. Coefficients of determination (R^2^) were calculated using the Past 4 software (version 4.16; Hammer and Harper, 2001).

## 3 Results and discussion

In this study, we adopted two complementary analytical techniques to cover different parts of *N. nuda* metabolome. A UHPLC-ESI-QToF-MS instrument, operating in both positive and negative ionization modes, enabled the chromatographic separation and identification of polar molecules in methanol extracts, primarily phenolics. It enabled us to comparatively and simultaneously analyze both glycosides and aglycones of iridoids in leaves and inflorescences. The solvent of choice for the present study was methanol as being vastly used for the extraction of both phenolics (e.g., Mišić et al., 2015; Hadi et al., 2017; Dienaitė et al., 2018; Aničić et al., 2021) and terpenoids (Aničić et al., 2024), including iridoid glycosides and volatile iridoid aglycones found in *Nepeta* species (Takeda et al., 1995; Petrova et al., 2022; Zaharieva et al., 2023).

### 3.1 Comparison of qualitative non-targeted phenolic profiles in *N*. *nuda* leaves and inflorescences

Totally 38 phenolic compounds were identified, including derivatives of hydroxybenzoic and hydroxycinnamic acids as well as flavonoid glycosides and aglycones (**Supplementary Table 2**).

**Figure 1:**
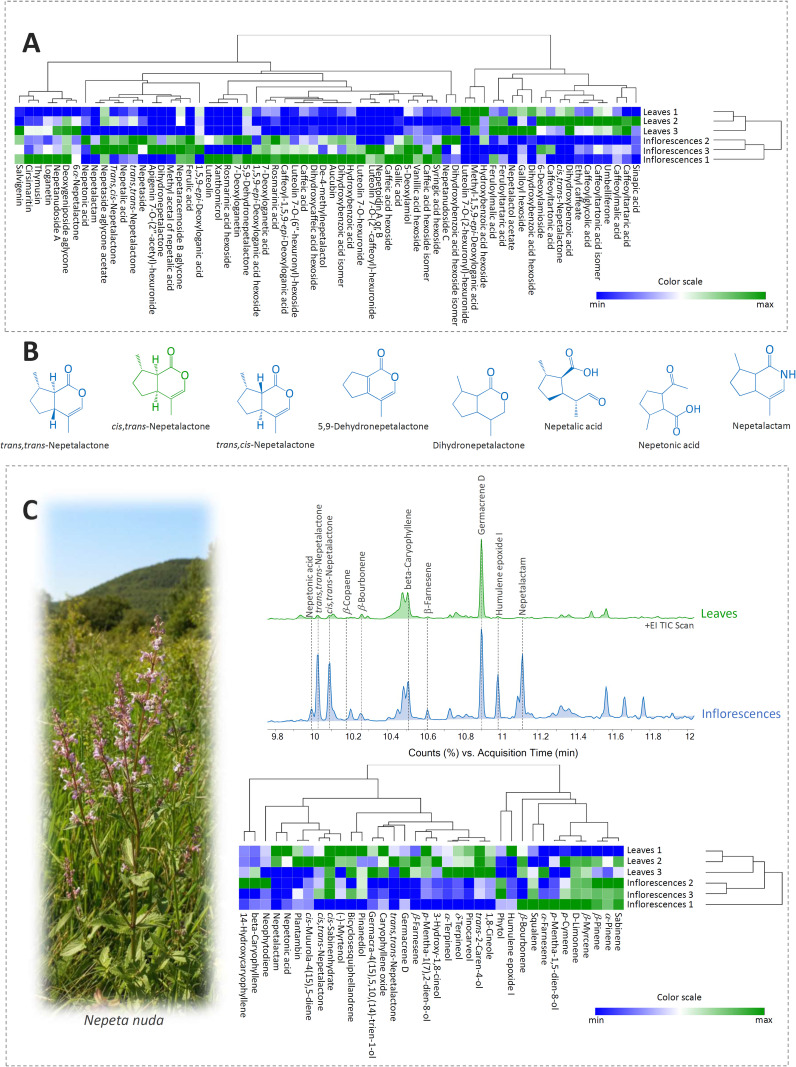
**(A)** Heatmap of the scaled QToF-MS data with the samples and compounds arranged according to the hierarchical cluster analysis (HCA), adopting the Spearman’s method of cluster agglomeration. **(B)** Chemical structures of the characteristic iridoid aglycones identified in *Nepeta nuda* methanol extracts of leaves and inflorescences. Iridoids predominating in inflorescences are represented in blue, while those prevalent in leaves are presented in green. **(C)** Representative TIC GC/MS chromatograms of methanol extracts of leaves and inflorescences. HCA plot of the scaled GC/MS data with populations and compounds arranged according to the Spearman’s method of cluster agglomeration. Plant photo denotes *N. nuda* individual captured on site (locality Straža, SE Serbia).

Nine derivatives of hydroxybenzoic acid were identified mainly as hexosides (**Supplementary Table 2**), giving a specific loss of 162 Da in MS^2^ spectra. All hydroxybenzoic acid derivatives were detected in leaves, while only one compound, dihydroxybenzoic acid (**3**), was not identified in the tested inflorescences (**Supplementary Table 2**). The majority of compounds belonging to the group of hydroxybenzoic acids were more abundant in inflorescences than in leaves of *N. nuda* except galloyl hexoside (**1**) and dihydroxybenzoic acid hexoside (**4**) (**Figure 1A**).

All nineteen compounds from the group of hydroxycinnamic acids were detected in inflorescences, whereas compounds **13**, **24**, **27**, and **28** were not found in leaves (**Supplementary Table 2**). Interestingly, esters of caffeic acid (**23**), malic acid (**25**), tartaric acid (**11** and **21**), tartronic acid (**12** and **16**), and glycolic acid (**14**) were more abundant in leaves than in inflorescences. The majority of other identified hydroxycinnamic acids were more abundant in inflorescences (**Figure 1A**). Compound **24** was not previously detected in any *Nepeta* species, but its MS^2^ spectra showed fragments typical for rosmarinic acid, which is characteristic for some *Nepeta* taxa (Mišić et al., 2015). This compound showed a neutral loss of 162 Da leading to the formation of a fragment ion at 359 *m/z* that corresponds to the exact mass of deprotonated rosmarinic acid. By studying other MS^2^ fragments, this compound was marked as rosmarinic acid hexoside.

All identified flavonoid compounds belong to the subgroup of flavones, which is typical for the genus *Nepeta* (Jamzad et al., 2003). The identified flavonoid aglycones were generally more abundant in inflorescences than in leaves. Hexuronides of luteolin (4 compounds) and apigenin (1 compound) were also recorded in the analyzed samples of *N. nuda* (**Supplementary Table 2**). Inflorescences were especially rich in these compounds. Flavones (mainly apigenin and luteolin derivatives) glycosylated with hexuronic (glucuronic) acid, often with another acyl residue (acetyl, coumaroyl, caffeoyl or similar), have been previously reported as constituents of several *Nepeta* species (Rabee et al., 2020). Compound **59** was identified as luteolin 7-*O*-(2”-caffeoyl)-hexuronide. In its MS^2^ spectra, ions were detected at 443 and 337 *m/z*, formed by the neutral loss of caffeic acid and luteolin, respectively (**Supplementary Figure 1**). This compound showed an unusual fragmentation pattern as its MS^2^ base peak was not the mass of luteolin aglycone (285 *m/z*) but corresponded to the mass of caffeic acid-H-H_2_O and it was previously identified in *Satureja biflora* aerial parts (Moghadam et al., 2015).

In the present study, we adopted the combination of analytical techniques previously reported to be convenient for establishing chemical characterization of *N. nuda* populations from the Central Balkans (Petrović et al., 2024). The mentioned study was focused on the chemical profiling of leaves dried in silica gel immediately after harvesting, while the samples used in this study were air-dried at room temperature until they reached a constant mass. This enabled us to compare and discuss the phytochemical composition of methanol extracts as influenced by drying procedure. Thus, vanillic acid was recorded only in silica gel-dried samples of *N. nuda* leaves (Petrović et al., 2024). Within the group of hydroxycinnamic acids, caffeic acid hexoside (**13**), ferulic acid (**27**), and nepetoidin A or B (**28**) were not present in air-dried leaves but were detected in inflorescences. The qualitative composition of flavonoids was also slightly perturbed, as luteolin (**61**) was recorded only in air-dried leaves, while chrysoeriol and acacetin were identified in leaves dried on silica-gel (Petrović et al., 2024).

### 3.2 Comparison of qualitative non-targeted iridoid profiles of *N. nuda* leaves and inflorescences

Twenty-seven iridoid compounds were identified in *N. nuda* inflorescences and leaves, 11 out of them being glycosides and 16 aglycones (**Supplementary Table 2**). One nitrogen analogue of nepetalactone, nepetalactam, was also recorded in *N. nuda* inflorescences. The presence of iridoid compounds was confirmed by the comparison with the available literature on *Nepeta* species and through the evaluation of their MS data. Some of the major iridoids identified in leaves and inflorescences of *N*. *nuda* are presented within **Figure 1B**.

Iridoid aglycones were mostly visible in the positive ionization mode due to their polarity, except for 7-deoxyloganetin, nepetalic and nepetonic acids (compounds **47**, **48**, and **50**, respectively), which gave more abundant peaks in the negative ionization mode. Among the identified iridoid aglycones, 7-deoxyloganetin (**47**) and dihydronepetalactone (**51**) were recorded in inflorescences only, while *cis*,*trans*-nepetalactone (**52**) and nepetalactol acetate (**53**) were characteristic for *N. nuda* leaves.

Nepetalactones and their derivatives were the most abundant iridoid aglycones in the analyzed *N. nuda* samples. This iridoid monoterpenoid can usually be found in the form of four diastereoisomers, three of them, *trans*,*trans*-, *cis*,*trans*-, and *trans*,*cis*-nepetalactone, being identified in the present study. Previous studies dealing with the phytochemical characterization of *N. nuda* reported all 4 diastereoisomers: *trans*,*trans*- (De Pooter et al., 1988; Handjieva et al., 1996; Petrović et al., 2024), *cis*,*trans*- (De Pooter et al., 1988; Kökdil et al., 1999; Kobaisy et al., 2005; Mancini et al., 2009; Narimani et al., 2017; Akbaba et al., 2021; Petrović et al., 2024), *trans*,*cis*- (Regnier et al., 1967; De Pooter et al., 1988; Mancini et al., 2009; Gkinis et al., 2010; Bozok et al., 2017; Narimani et al., 2017; Akbaba et al., 2021; Aćimović et al., 2022), and *ci*s,*cis*-nepetalactone (De Pooter et al., 1988; Kökdil et al., 1999; Kobaisy et al., 2005; Mancini et al., 2009; Bozari et al., 2013; Gormez et al., 2013; Zaharieva et al., 2023). In our study, *trans*,*trans*-nepetalactone was more abundant in inflorescences than in leaves (**Figure 1A**), while *cis*,*trans*-nepetalactone was recorded in leaves only. *trans*,*cis*-Nepetalactone was recorded in trace amounts in both leaves and inflorescences (**Supplementary Table 2**).

5,9-Dehydronepetalactone (**40**) is a dehydrogenated nepetalactone, isolated for the first time from *N. cataria* (Sastry et al., 1972). In *N. nuda*, this compound is primarily accumulated in inflorescences, while leaves contain considerably lower amounts (**Figure 1A**). This compound was previously reported in *N. nuda* (De Pooter et al., 1988; Kökdil et al., 1999; Petrović et al., 2024) and it was quantified in significant amounts in methanol extracts of *N. rtanjensis*, *N. argolica*, and *N. parnassica* (Mišić et al., 2015; Aničić et al., 2018, 2020, 2021). The content of 5,9-dehydronepetalactone in *N. teydea* EO was higher when it was prepared from flowering plants than from plants in the vegetative growth stage (Velasco-Negueruela et al., 1989), which is consistent with the findings of the present study.

Dihydronepetalactone (**51**) was identified in *N. nuda* inflorescences only (**Supplementary Table 2**). It is formed by nepetalactone hydrogenation (Feaster et al., 2009) and is known to be a common constituent of *N. cataria* (Regnier et al., 1967; Sharma and Cannoo, 2013; Sengupta et al., 2018; Handjeva et al., 1996; Patel et al., 2022).

Nepetalic acid (syn. 3α-hydroxy-4aα,7α,7aα-dihydronepetalactone, 3R-hydroxy-4aR,7R,7aR-dihydronepetalactone) can be synthetically converted into nepetalactone (Chauhan and Zhang, 2008), which marks this compound and plants rich in it as potentially valuable industrial resources. *N. nuda* is a good candidate as it accumulates nepetalic acid (**48**) in high amounts, especially in inflorescences (**Supplementary Table 2**, **Figure 1A**).

A derivative of nepetalic acid, its methyl isomer, nepetonic acid (2‚-[(1-methyl-2-al)-ethyl-5R-methylcyclopenta‚-carboxylic acid) (**50**), was identified in *N. nuda* samples in the present study (**Supplementary Table 2**). Methyl acetal of nepetalic acid (**54**) can be synthesized by nepetalic acid acylation (Scialdone and Liauw, 2008) and it was identified exclusively in inflorescences of *N. nuda*, adopting the UHPLC/QToF-MS technique (**Supplementary Table 2**).

Nepetalactam (**81**), classified under the group of tetrahydropyridine metabolites (lactams or cyclic amides), is a nitrogen analogue of (4aS,7S,7aR; *cis,trans*-)-nepetalactone previously identified in the essential oil of *N. cataria* (Handjeva et al., 1996; Patel et al., 2022), but also synthetized from nepetalic acid and nepetalactone (Eisenbraun et al., 1988; Chauhan et al., 2014). Interestingly, this compound was accumulated only in *N. nuda* inflorescences (**Supplementary Table 2**), indicating its possible contribution in plant-biotic interactions and reproductive strategy of this plant species. This compound was not identified in our previous study adopting a similar analytical approach (Petrović et al., 2024), most certainly since inflorescences were not chemically characterized.

Derivatives of iridoid glycosides were identified in the negative ionization mode (**Supplementary Table 2**), mostly showing [M–H]^–^ as a molecular ion, while adducts with formic acid ([M + HCOOH–H]^–^) were observed in three iridoid glycosides (**31**, **33**, and **34**). The majority of identified compounds from this group were present in both inflorescences and leaves. The exception was caffeoyl-1,5,9-*epi*-deoxyloganic acid (**39**), which was identified in *N. nuda* inflorescences only.

1,5,9-*epi*-Deoxyloganic acid was previously identified in many *Nepeta* species (Murai et al., 1984; Nagy et al., 1998; Aničić et al., 2021, including *N. nuda* (Kökdil et al., 1999; Petrova et al., 2022; Petrović et al., 2024). In the present study, 1,5,9- *epi*-deoxyloganic acid was detected in both leaves and inflorescences, being more abundant in the latter. Its three derivatives were also recorded. 1,5,9-*epi*-Deoxyloganic acid hexoside was very abundant in leaves and especially in inflorescences, while methyl-1,5,9-*epi*-deoxyloganic acid was found in leaves of *N. nuda*. To the best of our knowledge, caffeoyl-1,5,9-*epi*-deoxyloganic acid was not previously recorded in any *Nepeta* species, and in the present study it was identified in significant amounts only in inflorescences of *N. nuda*.

The most abundant compound from the subgroup of iridoid glycosides, compared by peak area, was nepetanudoside A (compound **34**). This compound, typical for *N. nuda* (Takeda et al., 1995; Petrović et al., 2024), was detected in both inflorescences and leaves but was more abundant in the reproductive organs (**Figure 1A**). The same trend was recorded for nepetanudoside C (**31**), which was previously reported for *N. nuda* (Takeda et al., 1996; Petrović et al., 2024).

Aucubin (**29**), present as a minor component in methanol extracts, was more abundant in inflorescences than in leaves of *N. nuda* and was previously reported for other *Nepeta* species, including *N. septemcrenata* (El-Moaty, 2010), *N. rtanjensis*, and *N. argolica* (Aničić et al., 2021). Lamiol derivative, 5-deoxylamiol (**32**), is an iridoid glycoside typical for *Lamium* species (Alipieva et al., 2003 and 2007) and has only recently been identified in *N. nuda* (Petrović et al., 2024). On the other hand, 6-deoxylamioside (**38**), which is here identified in both inflorescences and leaves, was previously reported for *L. amplexicaule* (Guiso and Martino, 1983).

To visualize the relations in metabolic fingerprints of *N. nuda* leaves and inflorescences, HCA was performed based on the Spearman’s method of cluster agglomeration, utilizing the relative quantification data (peak areas) (**Figure 1A**). These two plant organs are clearly diversified based on the composition of phenolics and iridoids, as the majority of the identified compounds are more abundant in inflorescences than in leaves. Compounds, on the other hand, are divided into two major clusters, based on their predominance in leaves or inflorescences.

### 3.3 Comparison of qualitative non-targeted profiles of volatile organic compounds in *N*. *nuda* leaves and inflorescences

In total, 36 VOCs were identified in methanol extracts of *N. nuda* inflorescences and leaves (**Table 1**).

**Table 1:** GC/MS profiling of methanol extracts of *Nepeta nuda* inflorescences and leaves.

No.	*t*_R_ (min)	Compound assignment	Chemical formula	Inflorescences	Leaves
Monoterpene hydrocarbons				1.24	4.62
**1G**	6.39	*α*-Pinene	C_10_H_16_	0.08	0.34
**2G**	6.82	Sabinene	C_10_H_16_	0.30	1.11
**3G**	6.87	*β*-Pinene	C_10_H_16_	0.49	1.91
**4G**	6.97	*β*-Myrcene	C_10_H_16_	0.21	0.74
**5G**	7.33	*p*-Cymene	C_10_H_14_	0.07	0.22
**6G**	7.37	D-Limonene	C_10_H_16_	0.09	0.30
Oxygenated monoterpenes				65.81	37.36
**7G**	7.41	1,8-Cineole	C_10_H_18_O	12.67	22.56
**8G**	7.73	*cis*-Sabinenhydrate	C_10_H_18_O	0.48	0.98
**9G**	8.11	*trans*-2-Caren-4-ol	C_10_H_16_O	0.06	0.05
**10G**	8.39	Pinocarveol	C_10_H_16_O	0.35	0.04
**11G**	8.60	*δ*-Terpineol	C_10_H_18_O	1.21	0.76
**12G**	8.78	*α*-Terpineol	C_10_H_18_O	1.86	0.92
**13G**	8.84	(-)-Myrtenol	C_10_H_16_O	0.19	0.27
**14G**	9.04	Pinanediol	C_10_H_18_O_2_	0.13	0.26
**15G**	9.16	3-Hydroxy-1,8-cineol	C_10_H_18_O_2_	0.14	0.09
**16G**	9.49	*p*-Mentha-1(7),2-dien-8-ol	C_10_H_16_O	0.11	0.07
**17G**	9.95	*p*-Mentha-1,5-dien-8-ol	C_10_H_16_O	0.03	0.28
**18G**	10.03	*trans*,*trans*-Nepetalactone	C_10_H_14_O_2_	44.05	2.57
**19G**	10.09	*cis*,*trans*-Nepetalactone	C_10_H_14_O_2_	4.52	8.53
Sesquiterpene hydrocarbons				20.74	32.84
**20G**	10.26	*β*-Bourbonene	C_15_H_24_	1.06	2.03
**21G**	10.51	beta-Caryophyllene	C_15_H_24_	2.07	7.03
**22G**	10.56	*cis*-Muurola-4(15),5-diene	C_15_H_24_	0.14	0.27
**23G**	10.61	*β*-Farnesene	C_15_H_24_	2.42	0.12
**24G**	10.66	Bicyclosesquiphellandrene	C_15_H_24_	0.10	0.09
**25G**	10.90	Germacrene D	C_15_H_24_	14.88	23.03
**26G**	10.94	*α*-Farnesene	C_15_H_24_	0.08	0.28
Oxygenated sesquiterpenes				1.69	1.49
**27G**	11.57	Caryophyllene oxide	C_15_H_24_O	0.88	0.75
**28G**	11.72	Humulene epoxide I	C_15_H_24_O	0.06	0.13
**29G**	12.04	14-Hydroxycaryophyllene	C_15_H_24_O	0.07	0.25
**30G**	12.14	Germacra-4(15),5,10(14)-trien-1-ol	C_15_H_24_O	0.61	0.25
**31G**	13.11	Platambin	C_15_H_26_O_2_	0.07	0.10
Diterpene hydrocarbons				0.11	0.62
**32G**	12.81	Neophytadiene	C_20_H_38_	0.11	0.62
Oxygenated diterpenes				1.84	19.02
**33G**	14.18	Phytol	C_20_H_40_O	1.84	19.02
Oxygenated triterpenes				0.19	1.90
**34G**	17.30	Squalene	C_30_H_5_0	0.19	1.90
Other compounds				8.04	2.00
**35G**	9.99	Nepetonic acid	C_9_H_14_O3	4.12	2.00
**36G**	11.12	Nepetalactam	C_10_H_15_NO	3.92	/

Values represent means of three biological replicates and are presented relatively in % (100% refers to the total peak area of the specific compound).

The representative GC/MS TIC chromatograms are shown in **Figure 1C**. These VOCs belong to the groups of monoterpenes (6 monoterpenes and 13 oxygenated monoterpenoids) and sesquiterpenes (7 sesquiterpenes and 5 oxygenated sesquiterpenoids). Nepetalactam (**36G**), recorded only in inflorescences, was not classified in any of the two groups. Monoterpenoids were more abundant in leaves, while inflorescences were richer in oxygenated monoterpenoids (**Table 1**). Among monoterpenoids, *β*-pinene (**3G**) and sabinene (**2G**) predominated in *N. nuda* leaves. 1,8-Cineole (**7G**) was more abundant in leaves, although a significant amount of this compound was recorded in inflorescences, as well. From the group of monoterpenoid iridoids, two nepetalactone stereoisomers were detected: *trans*,*trans*- (**18G**) and *cis*,*trans*-nepetalactone (**19G**), the former predominating in inflorescences and the latter in leaves. Nepetonic acid (**35G**) was also more abundant in inflorescences. Interestingly, *trans*,*cis*- isomer of nepetalactone and some of nepetalactone derivatives (5,9-dehydronepetalactone, dihydronepetalactone, nepetalic acid), previously identified by the UHPLC-ESI-QToF-MS technique, were not recorded in methanol extracts of *N. nuda* samples using the GC/MS analysis. This can be, at least partially, ascribed to differential chromatography conditions (UHPLC vs. GC) and huge differences in the sensitivities of mass spectrometers.

Sesquiterpenoids and oxygenated sesquiterpenoids were more abundant in leaves than in inflorescences of *N. nuda* (**Table 1**). Germacrene D (**25G**) and beta-caryophyllene (**21G**) were detected as the major sesquiterpenoids in leaves, followed by *β*-bourbonene (**20G**). In inflorescences, germacrene D (**25G**) was the most abundant compound as well but was followed by *β*-farnesene (**23G**) and beta-caryophyllene (**21G**). Oxygenated diterpene phytol (**33G**) and oxygenated triterpene squalene (**34G**) were by far more abundant in leaves of *N. nuda* than in inflorescences. In sum, inflorescences and leaves of *N. nuda* were clearly distinguished based on the content of teprenoids and related compounds and can therefore be considered as different chemotypes.

Only scarce literature data reported on the composition of *N. nuda* methanol extracts revealed by GC/MS characterization. Other studies primarily focused on analyzing essential oils composition and reported the following compounds as dominant components: caryophyllene oxide (Kökdil et al., 1998), camphor and 1,8-cineole (Kilic et al., 2011), caryophyllene (Alim et al., 2009), nepetalactone (Bozok et al., 2017; Handjieva et al., 1996; Kökdil et al., 1996), 1,8-cineole and 4a*α*,7*β*,7a*α*-nepetalactone (Mamadalieva et al., 2016), nepetalactone and germacrene (Gormez et al., 2013), 1,8-cineole (Chalchat et al., 1998), and 1,8-cineole and a mixture of nepetalactones and germacrene-D (De Pooter et al., 1988). It must be considered that these differences might arise from many factors, including plant origin, growth conditions, developmental stage, plant part analyzed, a subspecies or a cultivar in question, essential oils isolation procedure, and GC/MS conditions. A study by Zaharieva et al. (2023) revealed 4a*α*,7*β*,7a*α*-nepetalactone, 1,8-cineole/eucalyptol, and germacrene D as the most abundant volatile compounds in *N. nuda* plants grown under either *in vitro* or *ex vitro* conditions. Caryophyllene, *β*-ocimene, bicyclogermacrene, *β*-pinene, myrcene, and humulene were also found in quite fair quantities.

The relative GC/MS quantitative data (peak areas) of methanol extracts of *N. nuda* were subjected to HCA to visualize the phytochemical relations between inflorescences and leaves (**Figure 1C**). Leaves cluster separately from inflorescences on the HCA plot constructed based on the Spearman’s algorithm, confirming the clear diversification between these plant parts. Compounds are clustered based on their predominance in inflorescences or leaves.

### 3.4 Organ-specific variation in the quantitative content of major iridoids and phenolics among the Central Balkans’ populations of *N*. *nuda*

**Figure 2:**
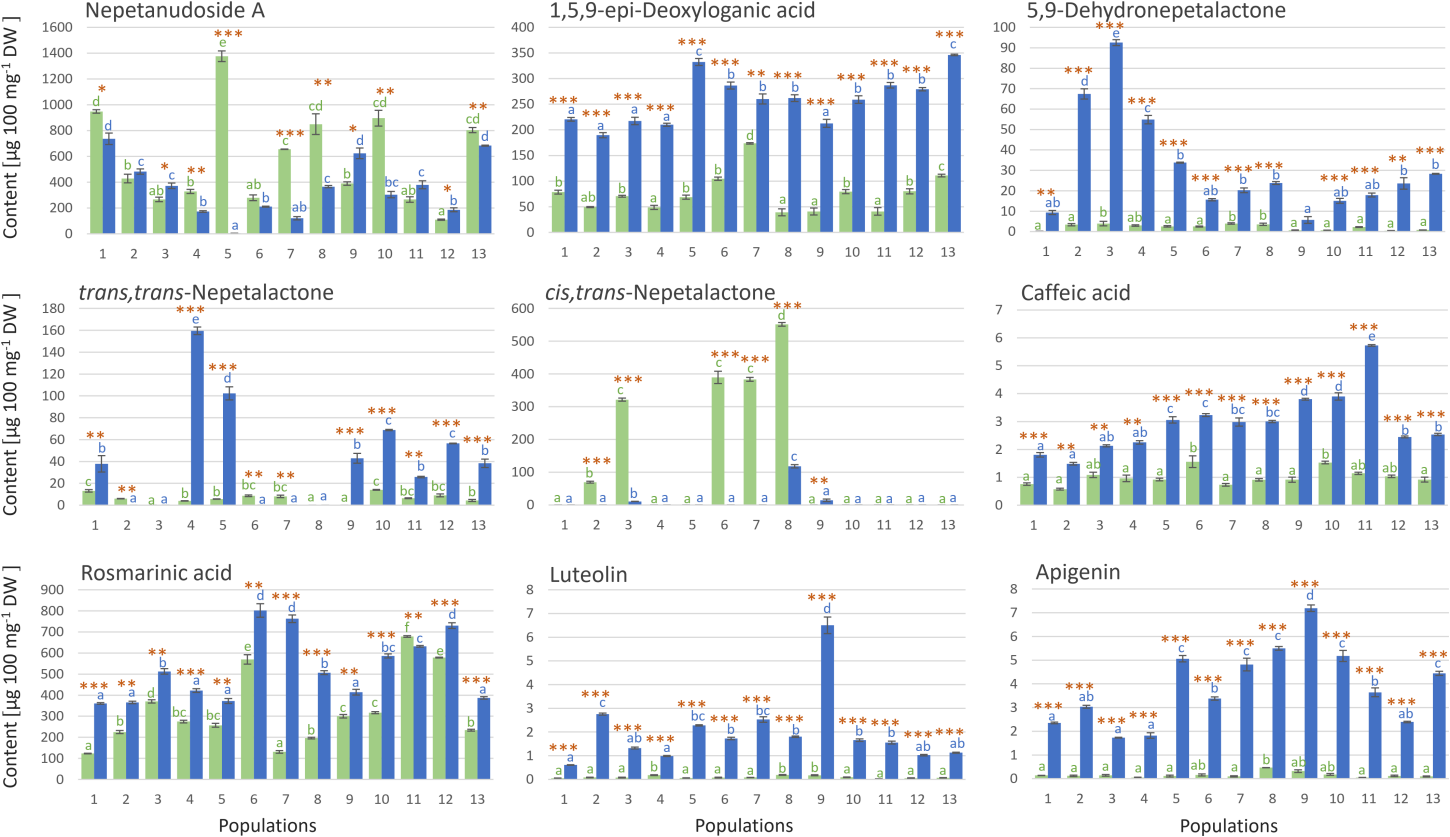
UHPLC/DAD/(±)HESI−MS^2^ quantification of targeted iridoids and phenolics in methanol extracts of *Nepeta nuda* leaves (green bars) and inflorescences (blue bars), in samples originating from 13 Central Balkan populations. SE of three biological replicates is presented. Values labeled with different letters, for leaves (green letters) and inflorescences (blue letters) independently, are significantly different (*p* < 0.05) according to the Tukey’s *post hoc* test of one way ANOVA. Orange asterisks above the bars denote significantly different values between leaves and inflorescences according to the t-test, *p*-values, *p < 0.05, **p < 0.01, ***p < 0.001. Abbreviations of *N*. *nuda* populations: 1-Mali Ljukten, 2-Brodica, 3-Debeli lug, 4-Rtanj, 5-Straža, 6-Židilje, 7-Vinatovača, 8-Vlasina, 9-Donji Krivodol, 10-Topli Do, 11-Janjska reka, 12-Balta Berilovac, 13-Gornje selo.

The UHPLC/DAD/(±)HESI−MS^2^ analysis was targeted towards totally 9 compounds belonging to the groups of iridoids (5 compounds) and phenolics (4 compounds), and the selection was based on previous studies reporting high abundance of these compounds in *N. nuda* (Mišić et al., 2015; Aras et al., 2016; Smiljković et al., 2018; Petrova et al., 2022; Petrović et al., 2024). From the group of iridoids, we selected three aglycones (*cis*,*trans*- and *trans*,*cis*-nepetalactone, and 5,9-dehydronepetalactone) and two glycosides (1,5,9-*epi*-deoxyloganic acid and nepetanudoside A). The most abundant phenolics in *N. nuda* were rosmarinic acid and caffeic acid (Mišić et al., 2015; Petrova et al., 2022), and these two phenolic acids were quantified along with two flavonoids from the group of flavones, luteolin and apigenin (**Figure 2**). The majority of the analyzed metabolites were more abundant in inflorescences than in leaves of *N. nuda*. Literature data support these findings, as inflorescences have generally higher constitutive levels of defensive metabolites than leaves (Brown et al., 2003; Damle et al., 2005; Smallegange et al., 2007), with some exceptions (Godschalx et al., 2016). On the other hand, the amounts of these compounds were variable among the 13 analyzed populations.

The results display obvious differentiation between leaves and inflorescences (**Figure 2**). The major iridoid glycoside, nepetanudoside A, was present in both inflorescences and leaves of *N. nuda*, and in populations 1, 4, 5, 7, 8, 10, and 13, the content of this compound was significantly higher in leaves than in inflorescences. Population 5 was especially rich in this compound. 1,5,9-*epi*-Deoxyloganic acid was more abundant in inflorescences than in leaves of *N. nuda*, and this implies to all analyzed populations (**Figure 2**). The highest amounts of this compound were recorded in samples belonging to the populations 5 and 13. Of the iridoid aglycones, *trans*,*trans*-nepetalactone and 5,9-dehydronepetalactone were more abundant in flowers than in leaves of *N. nuda*. The content of *trans*,*trans*-nepetalactone varied between populations, with the population 4 and 5 being especially rich in this compound. The highest amounts of 5,9-dehydronepetalactone were recorded in samples belonging to the populations 2, 3, and 4. Interestingly, *cis*,*trans*-nepetalactone was recorded in significant amounts in only 5 populations (2, 3, 6, 7, and 8), primarily in leaves (**Figure 2**).

**Figure 3:**
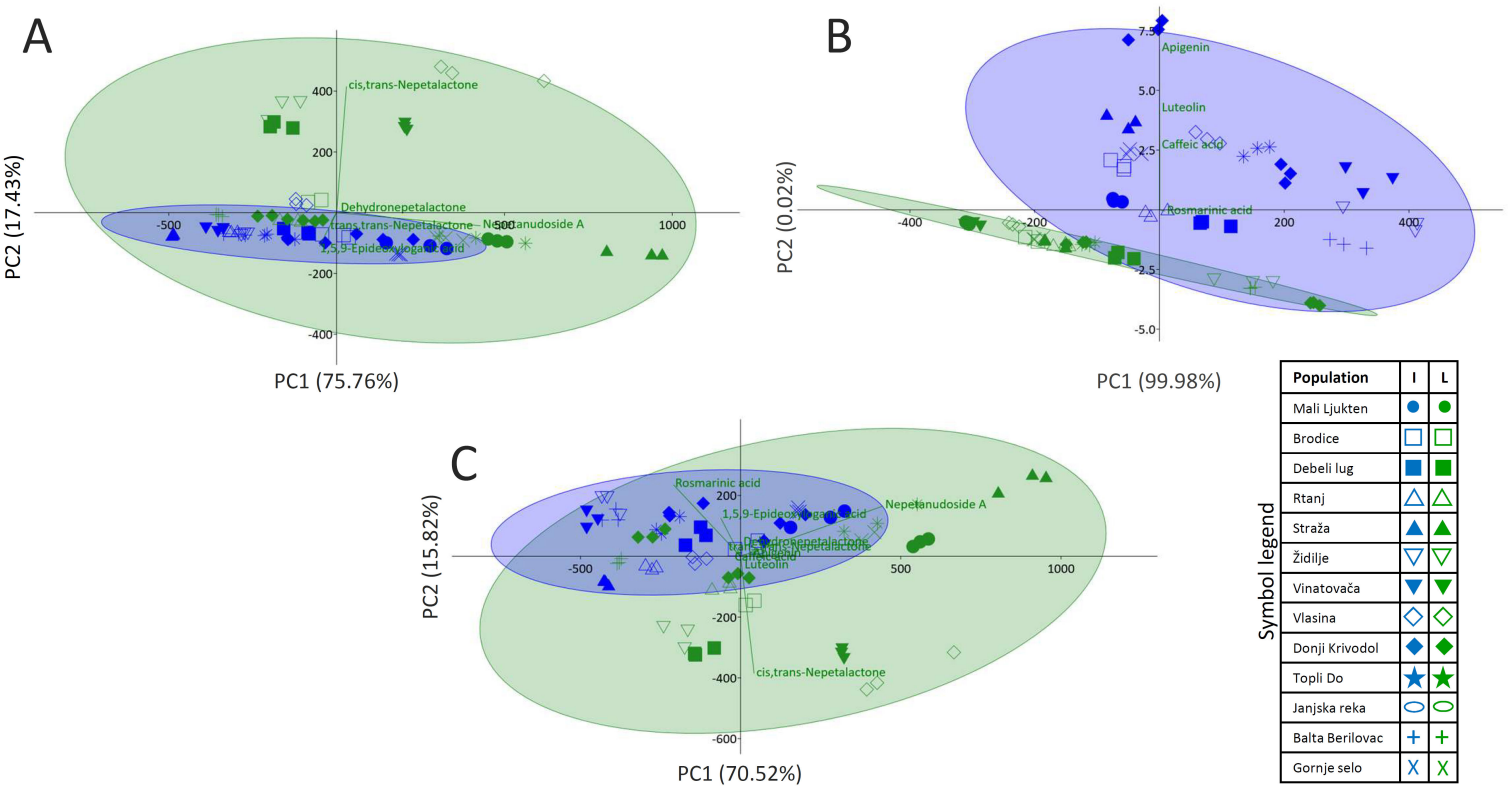
Principal component analysis (PCA) biplots constructed based on the UHPLC/DAD/(±)HESI−MS^2^ quantitative data, separately for iridoids **(A)** and phenolics **(B)**, and cumulatively for both groups of metabolites **(C)**. Participation of the variables (metabolites) in the first two PCs is indicated by the corresponding loading plots. Inflorescences are labeled with blue symbols while leaves are colored green.

To visualize variations in the quantitative composition of the major iridoids in inflorescences and leaves of 13 N*. nuda* populations, PCA was performed adopting the Ward’s method of data agglomeration (**Figure 3A**). The major contributors to the diversification of samples along the PC1 explaining 75.76% of the total variability are nepetanudoside A, *cis*,*trans*-nepetalactone, and 1,5,9-*epi*-deoxyloganic acid. As for the PC2, which contributes to the total variability with 17.43%, *cis*,*trans*-nepetalactone and 1,5,9-*epi*-deoxyloganic acid are the major contributors to the diversification along this component. Samples of *N. nuda* leaves are more scattered along PC1 than samples of inflorescences, implying higher uniformity in the accumulation of the targeted compounds by inflorescences.

Among phenolics, rosmarinic acid was by far the most abundant compound in the analyzed samples. It was recorded in considerable amounts in both leaves and inflorescences of *N. nuda*. Its amounts in inflorescences reached ∼802 µg 100 mg^-1^ dry weight (DW) (population 6), while in leaves ∼678 µg 100 mg^-1^ DW was recorded in samples from the population 11 (**Figure 2**). In the majority of the analyzed populations, the amounts of this compound were slightly higher in inflorescences than in leaves. Caffeic acid, luteolin, and apigenin were also more abundant in inflorescences than in leaves in all analyzed populations. Population 9 was characterized by the highest amounts of luteolin (6.50 µg 100 mg^-1^ DW) and apigenin (7.19 µg 100 mg^-1^ DW), while the highest amounts of caffeic acid were recorded for the population 11 (**Figure 2**).

When PCA was performed with quantitative data for phenolics only, it was obvious that samples of leaves and inflorescences were clearly diversified along PC2 (**Figure 3B**). The major contributors to the diversification along PC1, which explains 99.98% of the total variability, and along PC2 (0.02%), are apigenin and luteolin. The third contributor is caffeic acid. Results imply that phenolics content is more conserved in leaves than in inflorescences.

PCA plot was further constructed based on the quantitative data for both iridoids and phenolics (**Figure 3C**). PC1 explains 70.52% of the total variability, while PC2 is responsible for 15.82% of the variability. The major contributors to the diversification along PC1 are nepetanudoside A, rosmarinic acid, and *cis*,*trans*-nepetalactone. The same compounds, but with perturbed order, are the major factors that diversify samples along PC2.

### 3.5 Molecular background of iridoid biosynthesis in *N*. *nuda* inflorescences and leaves

We further aimed to get deeper into the molecular background of the organ-specific iridoid diversity in *N. nuda*. To exclude the effect of genotype and environmental conditions on the chemical phenotype, we performed clonal propagation of a single genotype originating from the population 3 (abbreviation DL1). Plantlets having the uniform genetic background were acclimatized and grown under controlled greenhouse conditions. Samples of leaves and inflorescences were collected from fully-flowering plants, in three biological repetitions.

**Figure 4:**
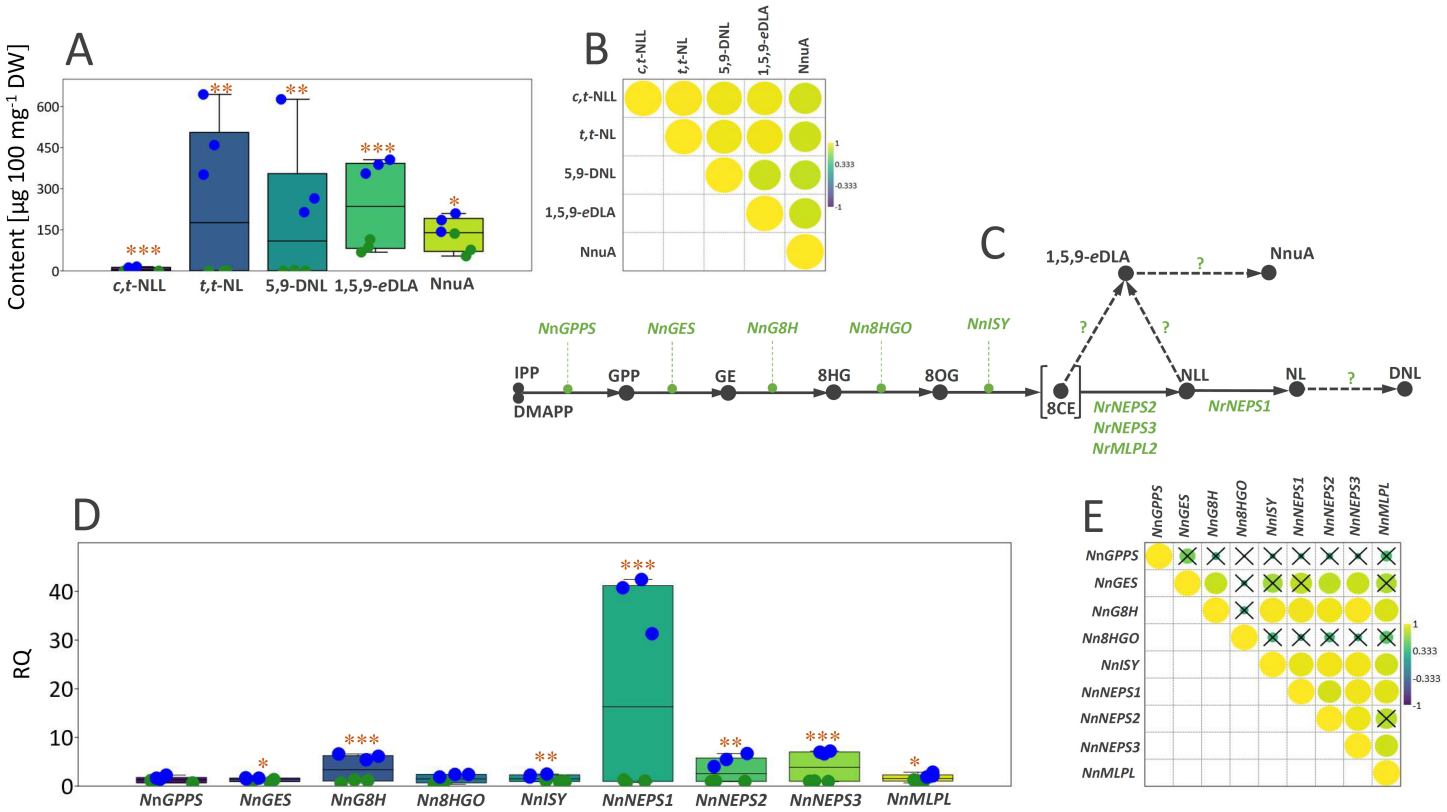
**(A)** Box-plot diagram depicting the distribution of the content of three iridoid aglycones (*c*,*t*-NLL, *t*,*t*-NL, 5,9-DNL) and two iridoid glycosides (1,5,9-*e*DLA and NnuA) in leaves (green dots) and inflorescences (blue dots) of *Nepeta nuda* grown under greenhouse conditions. **(B)** Pearson’s correlations based on the content of five major iridoids in *N. nuda* leaves and inflorescences represented by the intensity of yellow (positive correlations) or blue (negative correlations), as indicated on the color scale. **(C)** A proposed iridoid metabolic pathway in *N. nuda*. Unknown enzymes of the biosynthetic pathway are marked with the question mark. **(D)** Box-plot diagram depicting the distribution of the transcript levels of nine iridoid biosynthesis-related genes in leaves (green dots) and inflorescences (blue dots) of *N. nuda*. **(E)** Pearson correlations based on the expression level of nine biosynthetic genes in *N. nuda* leaves and inflorescences. Non-significantly correlated values are crossed (p > 0.05). IPP, isopentenyl pyrophosphate; DMAPP, dimethylallyl pirophosphate; GPP, geranyl pyrophosphate; GE, geraniol; 8HG, 8-hydroxygeraniol; 8OG, 8-oxogeranial; 8CE, 8-oxocitronellyl enolate; NLL, nepetalactol; NL, nepetalactone; DNL, dehydronepetalactone; *c*,*t*-NLL, *cis*,*trans*-nepetalactol; *t*,*t*-NL, *trans*,*trans*-nepetalactone; 5,9-DNL, 5,9-dehydronepetalactone; 1,5,9-*e*DLA, 1,5,9-*epi*deoxyloganic acid; NnuA, nepetanudoside A; NnGPPS, geranyl diphosphate synthase; NnGES, geraniol synthase; NnG8H, geraniol 8-hydroxylase; Nn8HGO, 8-hydroxygeraniol oxidoreductase; NnISY, iridoid synthase; NnNEPS, nepetalactol-related short-chain dehydrogenase; NnMLPL, major latex protein-like enzyme. Asterisks denote significantly different values according to the *t* test (*p < 0.05, **p < 0.01, ***p < 0.001).

Targeted metabolic profiling of five iridoids (*cis*,*trans*-nepetalactol, *trans*,*trans*-nepetalactone, 5,9-dehydronepetalactone, 1,5,9-*epi*-deoxyloganic acid, and nepetanudoside A) in leaves and inflorescences of greenhouse-grown *N. nuda* in parallel with co-expression analysis of iridoid biosynthesis-related genes, revealed the molecular background of their organ-specific composition. As expected, all the quantified iridoids were more abundant in inflorescences than in the leaves of *N. nuda* plants (**Figure 4A**). The difference in the abundance of specific iridoids between the two plant organs was the most pronounced for 1,5,9-*epi*-deoxyloganic acid and *cis*,*trans*-nepetalactol, the latter being recorded only in inflorescences. The correlation analysis based on the quantitative data revealed significant positive correlations for the majority of comparisons (**Figure 4B**). These results are in accordance with the study of Gomes et al. (2024), which associated the highest concentrations of nepetalactones, nepetalic acid and nepetalactam with the floral-bud and partial-flowering stages of *N. cataria*.

The iridoid biosynthetic pathway in *Nepeta* species has been extensively researched in the past, and it is believed that nepetalactol is the common precursor of all other iridoids in these species. The biosynthesis of nepetalactol involves a series of enzymatic steps catalyzed by geranyl diphosphate synthase (GPPS), geraniol synthase (GES), geraniol 8-hydroxylase (G8H), 8-hydroxygeraniol oxidoreductase (8HGO), iridoid synthase (ISY), NAD-dependent nepetalactol-related short-chain-dehydrogenase/reductase (NEPS), and major latex protein-like (MLPL) enzymes (**Figure 4C**). In *Nepeta* species, ISYs are mainly responsible for the stereoselective 1,4-reduction of 8-oxogeranial (8OG) to uncyclized and reactive 8-oxocitronellyl enol (8CE) and for determining the stereochemistry of C7 (Sherden et al., 2018; Lichman et al., 2019b and 2020; Hernaíndez Lozada et al., 2022). Enolate intermediate 8-oxocitronellyl enol in *Nepeta* is cyclized by NEPSs (Lichman et al., 2019a, 2019), but can also undergo a spontaneous cyclization to produce predominately *cis*,*trans*- steroisomer of nepetalactol (Hernaíndez Lozada et al., 2022). Although the upstream parts of the pathway have been well described, the downstream parts that are leading to various derivatives of nepetalactone, as well as those of the iridoid glycosides branch, remain largely unclear.

By BLAST-searching for the available *N. nuda* transcriptomes, candidates for 9 iridoid biosynthesis-related genes (*NnGPPS*, *NnGES*, *NnG8H*, *Nn8HGO*, *NnISY*, *NnNEPS1*, *NnNEPS2*, *NnNEPS3*, and *NnMLPL*) were identified and their expression profiles were comparatively analyzed in *N. nuda* leaves and inflorensces to more deeply investigate the molecular background of their variation between the two organs and to point to the key determinants of the organ-specific iridoid metabolism in *N. nuda*. The expression levels of *NnGPPS* and *Nn8HGO*, two genes of the early biosynthetic pathway (EBGs), did not significantly differ between leaves and inflorescences (**Figure 4D**). However, *NnGES*, *NnG8H*, and *NnISY* displayed around 1.6-, 6-, and 2.2-fold higher transcript levels in inflorescences than in leaves, respectively. Interestingly, genes responsible for the steps directly preceding the formation of nepetalactol and nepetalactone (*NnNEPS1*, *NnNEPS2*, *NnNEPS3*, and *NnMLPL*) showed significantly higher expression level in inflorescences than in leaves (**Figure 4D**). In the case of *NnNEPS1*, which belongs to the subgroup of NEPS oxidases and is responsible for converting nepetalactol into nepetalactone, around 40-fold higher expression level was recorded in inflorescences than in leaves. The NEPS oxidase (*NnNEPS1*) identified in the transcriptomes of *N. nuda* leaves, that corresponded to the *N. mussinii* NEPS1, has been reported to have *cis*,*trans*-nepetalactol dehydrogenase activity (Lichman et al., 2019a). *NnNEPS2* is a homologue of *N. sibirica* NEPS2 (*Ns*NEPS2), which acts both as a *cis*-*trans* cyclase and an oxidase (Lichman et al., 2019a; Hernaíndez Lozada et al., 2022). *NnNEPS3* is a homologue of *N. mussinii* NEPS3 displaying a specific 7S-*cis*,*cis*-nepetalactone cyclase activity (Lichman et al., 2019a). The composition, expression level, and ratio of NEPS enzymes are responsible for setting the stereochemistry of nepetalactones (Lichman et al., 2019a), which is most certainly the case in *N. nuda* as well. The results indicate that *NnG8H* and the 3 *NEPSs* might serve as the regulatory points, determining the flux through the iridoid pathway and thus the level of iridoid biosynthesis and accumulation in different organs. Hence, the high level of nepetalactone and other iridoids in inflorescences is, most likely, the result of upregulated expression of *NnG8H*, *NnNEPS1*, *NnNEPS2*, and *NnNEPS3* in these organs. The majority of correlations observed between the analysed biosynthetic genes were positive and statistically significant, which suggests the regulation of iridoid biosynthesis in different organs at the transcriptional level (**Figure 4E**). The exception were *NnGPPS* and *Nn8HGO*, which displayed no significant correlations with other genes. Conversely, the expression of *NnGES* was significantly positively correlated with that of *NnG8H*, *NnNEPS2*, and *NnNEPS3*.

Some previous studies dealing with *Nepeta* species revealed organ-specific expression patterns of the iridoid biosynthesis-related genes (Aničić et al., 2018, and 2024, Lichman et al., 2020). Closed buds and open flowers of *N. cataria* and *N. mussinii* were more enriched with transcripts of NEPSs, G8H, and ISY than mature leaves (Lichman et al., 2020). Among NEPS enzymes, NEPS1 displayed the highest expression level in open flowers and closed buds of *N. cataria*, while NEPS5 transcripts were the most abundant in closed buds of *N. mussinnii* (Lichman et al., 2020). This is in accordance with the findings of the present study, as *NnNEPS1* displayed a significantly higher expression level in inflorescences than in mature leaves of *N. nuda* and more transcript levels than the other two NEPSs (*NnNEPS2* and *NnNEPS3*). Although we did not separately assessed closed buds from open flowers, the analyzed inflorescences contained flowers in all development stages.

The expression of iridoid biosynthesis-related genes is coordinately regulated and well correlated with metabolite pools in mature leaves and inflorescences (**Table 2**), supporting the hypothesis that the organ-specific biosynthesis of iridoids is controlled at the transcriptional level. In other words, gene transcripts of targeted genes were more abundant in inflorescences, where the majority of targeted iridoids accumulated. The exceptions are *NnGPPS*, *NnGES*, and *Nn8HGO*, as their expression levels were not significantly correlated with the content of targeted metabolites. The majority of significant correlations were observed between the three *NEPSs* and *G8H* on one hand and iridoid aglycones (5,9-dehydronepetalactone, *cis*,*trans*-nepetalactol, and *trans*,*trans*-nepetalactone) on the other. The most significant correlations were observed between the expression of *NnNEPS1* and *NnNEPS3* and the three iridoid aglycones. *NnNEPS1* was also positively correlated with the content of quantified iridoid aglycones, nepetanudoside A and 1,5,9-*epi*-deoxyloganic acid (**Table 2**), indicating possible involvement of this NEPS oxidase in the formation of iridoid glycosides. These speculations should be further investigated.

**Table 2:** Correlations (*R* ^2^) between quantitative metabolomics data (for 5 selected iridoid compounds) and qPCR expression data for 9 iridoid biosynthesis-related genes in the analyzed samples of *Nepeta nuda* (*n*= 324).

Genes	Metabolites				
	Nepetanudoside A	1,5,9-*epi*-Deoxyloganic acid	Dehydronepetalactone	*c*,*t*-Nepetalactol	*t*,*t*-Nepetalactone
***NnGPPS***	0.05099	0.01171	0.02422	0.01530	0.02615
***NnGES***	0.40227	0.69953	0.62632	0.73876	0.75092
***NnG8H***	0.49685	0.92993*	0.65089**	0.76221**	0.84667**
***Nn8HGO***	0.14440	0.07399	0.05780	0.01432	0.04141
***NnISY***	0.47210	0.86730	0.62146	0.66582**	0.77429**
***NnNEPS1***	0.50028**	0.91788**	0.48505***	0.69303***	0.75252***
***NnNEPS2***	0.59176	0.89129	0.84279	0.81969**	0.90995**
***NnNEPS3***	0.61061*	0.97990**	0.70291***	0.84138**	0.90471***
***NnMLPL***	0.27544	0.67000	0.27289	0.38925	0.48010*

*R* ^2^, coefficient of determination; n, total number of comparisons; ***, **, * – significance levels at *p* < 0.001, *p* < 0.01, and *p* < 0.05, respectively.

### 3.6 Possible ecological implications of iridoids’ composition in *N*. *nuda* inflorescences and leaves

Due to a variety of important roles that iridoids play in plant-biotic interactions, we here discuss possible ecological implications of their organ-specific composition in *N. nuda*. Data show that *N. nuda* leaves exhibit greater diversity in terms of the targeted phenolics’ and iridoids’ content, and that phytochemical composition of inflorescences is more conserved among the analyzed populations. Plants likely invest more resources in the constitutive production of floral metabolites functioning in plant-biotic interactions (i.e., plant-pollinator, plant-pathogen, and plant-herbivore interplay), rather than in inducible production. It was previously hypothesized that constitutive resistance predominates in flowers (McCall and Irwin, 2006; Zangerl, 2003). Floral chemistry influences the interactions of plants with pollinators and other species and maximizes the fitness of both plants and flower visitors (Palmer-Young et al., 2019; Kessler and Kalske, 2018). The defense mechanisms of reproductive tissues, such as flowers, are crucial for plant reproduction, and their defensive strategies may differ from those of leaves, which have the primary function of fixing CO_2_ during photosynthesis. According to Pichersky and Gershenzon (2002), floral volatiles serve as attractants for species-specific pollinators, whereas the volatiles emitted from vegetative parts, especially those released after herbivory, appear to protect plants by deterring herbivores and by attracting their enemies. Flowers are essentially heterotrophic as they heavily rely on organic molecules synthesized in leaves and roots. However, they biosynthesize and accumulate complex molecules that sustain plant interactions with animal pollinators and pathogens, play roles in plant fertilization, embryo development, and the initial stages of fruit and seed set.

The chemical composition of colorful long-lasting flower spikes of *N. nuda* is a key determinant of their color, odor, and overall function in plant-biotic interactions. *Nepeta* flowers with their visual, chemical, and nutritional traits are very attractive to honeybees and other pollinators. Honeybees are the main pollinators of *N. nuda* (Bozek, 2003). The most important visitors of flowers of *N. cataria*, a species sympatric to *N. nuda*, are honeybees, solitary bees, and bumblebees (Cruden and Miller-Ward, 1981; Sih and Baltus, 1987), but also flies and butterflies (Aizen and Harder, 2009), thus it may be presumed that *N. nuda* is visited by similar groups of pollinators, as these two species share similar phytochemical profiles.

The increased content of iridoids in the reproductive organs of *N. nuda* might indicate their functional involvement in complex plant-biotic interactions. It is well documented that iridoids function as general toxicants, growth and reproductive inhibitors, repellents or oviposition-deterrents (Bernier et al., 2005; Birkett et al., 2011; Sparks et al., 2018; Reichert et al., 2019; reviewed in Formisano et al., 2011; Süntar et al., 2018). Two groups of iridoids present in *Nepeta* plants, volatile aglycones and glycosylated iridoids, most likely provide a two-level chemical defense, the former acting as repellents and/or pollinator attractants, and the latter contributing as deterrents. Both subgroups of iridoids also provide antimicrobial protection.

Constituents of *Nepeta* species, such as nepetalactone, dihydronepetalactone, and nepetalactol, are proven repellents against insects, such as mosquitos (*Aedes*, *Anopheles*, *Culex*), cockroaches (*Periplaneta*, *Blattella*), ticks (*Rhipicephalus*, *Ixodes*), flies (*Stomoxys*, *Musca*), mites (*Dermanyssus*, *Dermatophagoides*), and termites (*Reticulitermes*) (Bernier et al., 2005; Birkett et al., 2011; reviewed in Formisano et al., 2011; Sparks et al., 2018; reviewed in Süntar et al., 2018; Reichert et al., 2019). Dihydronepetalactone is reported to be two times more active than commercial N,N-diethyl-3-methylbenzamide (DEET) (Feaster et al., 2009). Notably, nepetalactone and nepetalactol mediate plant-insect interactions, but are also produced by a number of insects, most notably aphids, acting as sex pheromones (Fernández-Grandon and Poppy, 2015). In addition, dihydronepetalactones are the components of defensive secretions of some ant species (Cavill and Clark, 1967). As reported by Harney et al. (1978), nepetalic acid, along with nepetalactone and catnip essential oil, had a depressant effect in mice when administered intraperitoneally.

On the other hand, compounds from the group of iridoid glycosides present in *Nepeta* species, less volatile and more stable than nepetalactones and their derivatives, have rarely been investigated for their interaction with insects and generally for their contribution to the overall defense strategies. 1,5,9-*epi*-Deoxyloganic acid has been scarcely investigated for its biological activities, thus, only studies dealing with its antimicrobial and anti-inflammatory potential (Aničić et al., 2021), as well as enzyme inhibition and DNA protection activities (Başar et al., 2023) are presented in the literature. To the best of our knowledge, no data about its role in plant-biotic interactions are available. Despite only scarce information about the bioactivities of the major iridoid glycosides of *N. nuda*, plenty is known about some iridoid glycosides present as minor constituents. Such an example is aucubin, which displays potent insecticidal activity against various insects (Vivekanandhan et al., 2022, 2023) and antifungal activity (Marak et al., 2002). Comprehensive literature search revealed that the roles of nepetenudoside A and C, as well as of nepetaside, 5-deoxylamiol, and 6-deoxylamioside in plant-biotic interactions have not been previously documented. Due to their physico-chemical properties, iridoid glycosides of *Nepeta* sp. might provide additional values in the development of novel biopesticides.

The composition of floral volatiles, a chemically diverse group of plant metabolites with multiple functions, is shaped by environmental, ecological and evolutionary factors (Dötterl and Gershenzon, 2023). Although iridoids are by far the most abundant group of terpenes in *Nepeta* species, their synergistic effects with other terpenes must not be ruled out. The unique metabolome of each individual plant constitutes the defense machinery against biotic environmental factors and determines its success in the changing environment. Several reports showed insecticidal and repellent activity of eucalyptol, which is abundant in both leaves and inflorescences of *N. nuda*. Eucalyptol induces intoxication and has a prominent effect on locomotor activity, knock-down, and repellence on nymphs of *Triatoma infestans* and *Rhodnius prolixus* (Moretti et al., 2015). It also displays repellence against a number of insects, including house fly (Sukontason et al., 2004), *Periplaneta americana* (Scriven and Meloan, 1984), and *Aedes aegypti* (Klocke et al., 1987). On the other hand, eucalyptol is attractive to honey bees and euglosine bees (Effmert et al., 2005).

It was previously reported that the content and ratio of sesquiterpenoids *β*-farnesene and germacrene D in flowers of *Tanacetum vulgare* modulated both defense and pollination (Li et al., 2019). Aphid alarm pheromone *β*-farnesene is the key volatile cue responsible for specific attraction of beetles and avoidance by aphids at the early flowering stages (Li et al., 2019). As these terpenoids are also found in *N. nuda* inflorescences and leaves, it could be hypothesized that they can play important roles in plant-biotic interactions.

### 3.7 Molecular background of the biosynthesis of phenolics in *N*. *nuda* inflorescences and leaves

**Figure 5:**
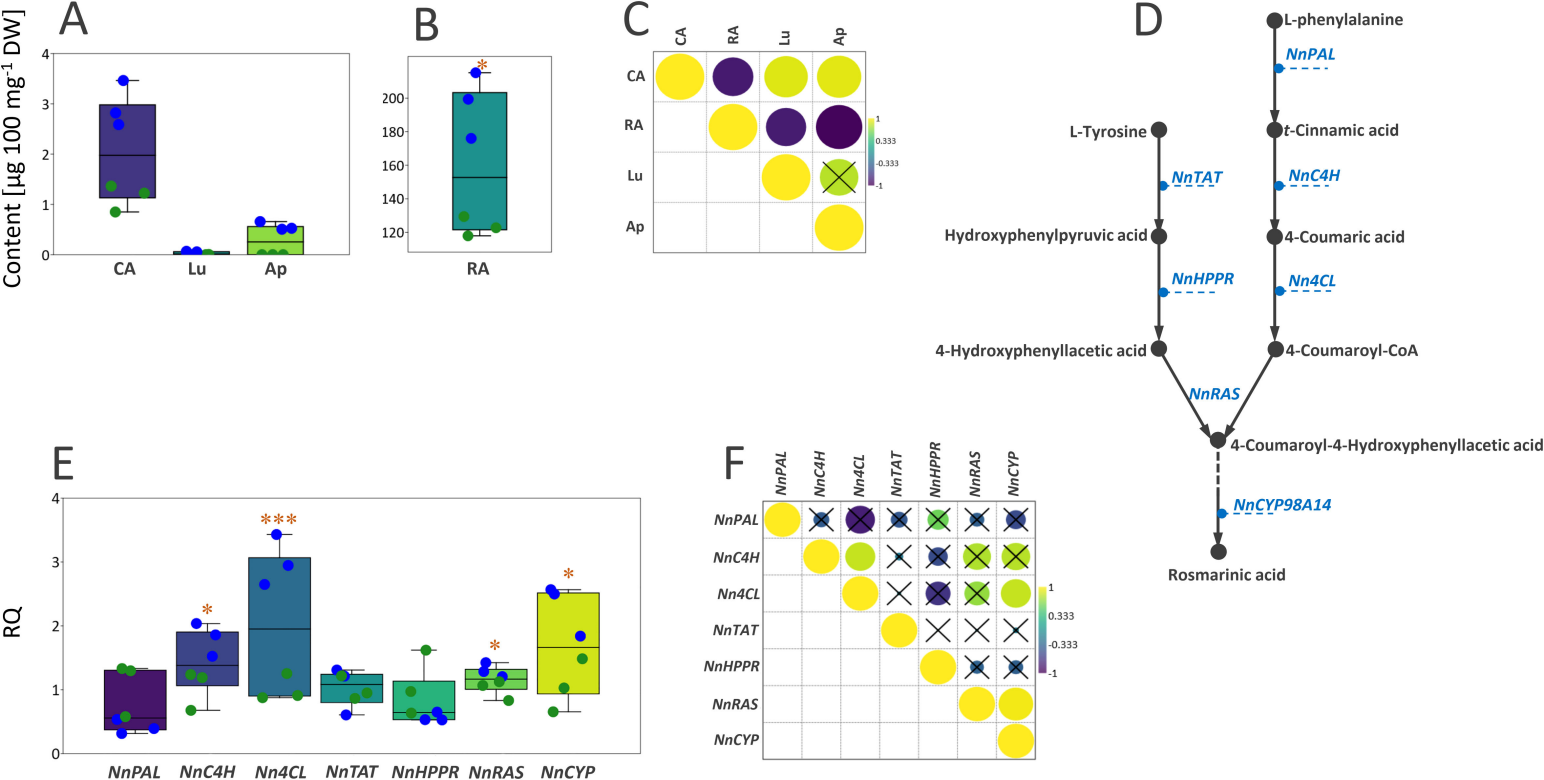
**(A)** Box-plot diagram depicting the distribution of the content of one phenolic acid (CA) and two flavonoids (Ap and Lu) in leaves (green dots) and inflorescences (blue dots) of *N. nuda* grown under greenhouse conditions. **(B)** RA is presented by a separate box-plot diagram. **(C)** Pearson’s correlations based on the content of four major phenolics in *N. nuda* leaves and inflorescences, represented by the intensity of yellow (positive correlations) or blue (negative correlations), as indicated on the color scale. Non-significantly correlated values are crossed (p > 0.05). **(D)** A proposed biosynthetic pathway of rosmarinic acid in *N. nuda*. **(E)** Box-plot diagram depicting the distribution of the transcript levels of seven rosmarinic acid biosynthesis-related genes in *N. nuda* leaves and inflorescences, represented by the intensity of yellow (positive correlations) or blue (negative correlations), as indicated on the color scale. **(F)** Pearson’s correlations based on the expression level of seven genes of the rosmarinic acid biosynthetic pathway in *N. nuda* leaves (green dots) and inflorescences (blue dots). Non-significantly correlated values are crossed (p > 0.05). CA, caffeic acid; RA, rosmarinic acid; Ap, apigenin; Lu, luteolin; PAL, phenylalanine ammonia-lyase; C4H, cinnamic acid 4-hydroxylase; 4CL, 4-coumaric acid CoA-ligase; TAT, tyrosine aminotransferase; HPPR, hydroxyphenylpyruvate reductase; RAS, 4-coumaroyl-CoA:4′-hydroxyphenyllactic acid 4-coumaroyltransferase; CYP, cytochrome P450-dependent monooxygenase. Asterisks denote significantly different values according to the *t* test (*p < 0.05, ***p < 0.001).

As expected, rosmarinic acid was by far the most abundant phenolic compound in leaves and inflorescences of *N. nuda* grown under greenhouse conditions (**Figures 5A, B**). Other phenolics quantified in samples were present in much lower amounts. All analyzed compounds were found in significantly higher amounts in inflorescences than in leaves. The amount of rosmarinic acid was significantly negatively correlated with those of caffeic acid, luteolin, and apigenin (**Figure 5C**). The only non-significant correlation was observed between luteolin and apigenin.

The biosynthesis of rosmarinic acid (**Figure 5D**) involves phenylpropanoid- and tyrosine-derived branches that use the amino acids L-phenylalanine and L-tyrosine as precursors, respectively (Matsuno et al., 2002; Petersen et al., 2009; Wang et al., 2017; Trócsányi et al., 2020; Suthar et al., 2021) and it proceeds by activating the following enzymes: phenylalanine ammonia-lyase (PAL), cinnamic acid 4-hydroxylase (C4H), 4-coumaric acid CoA-ligase (4CL), tyrosine aminotransferase (TAT), hydroxyphenylpyruvate reductase (HPPR), “rosmarinic acid synthase” (RAS; 4-coumaroyl-CoA:4′-hydroxyphenyllactic acid 4-coumaroyltransferase), and cytochrome P450-dependent monooxygenase. We here analyzed the expression profiles of 7 genes related to the biosynthesis of rosmarinic acid (*NnPAL*, *NnC4H*, *Nn4CL*, *NnTAT*, *NnHPPR*, *NnRAS*, and *NnCYP*) in *N. nuda* leaves and inflorescences. Looking at their enzymatic activities involved in this biosynthetic pathway, only two of them might be specific for rosmarinic acid biosynthesis, *NnRAS* and *NnCYP*. These two genes showed significantly higher expression levels in inflorescences than in leaves (**Figure 5E**), which is in accordance with a higher content of rosmarinic acid in these organs. Enzymes belonging to the general phenylpropanoid pathway (*NnPAL*, *NnC4H*, and *Nn4CL*) provide precursors for the formation of other metabolites such as lignin and other groups of phenolic compounds (e.g., flavonoids, anthocyanins). Transcript levels of *NnC4H* and *Nn4CL* were higher in inflorescences than in leaves, while *NnPAL* did not significantly differ between the two organs (**Figure 5E**). TAT and HPPR are also primary enzymes, and their activity results in the formation of precursors in the biosynthesis of tocopherols and plastoquinones. These two genes did not significantly differentially express between leaves and inflorescences of *N. nuda* (**Figure 5E**). The only significant correlations were positive correlations between the expression patterns of the analyzed rosmarinic acid-related biosynthetic genes: *Nn4CL* and *NnC4H*, *NnCYP* and *Nn4CL*, and *NnCYP* and *NnRAS* (**Figure 5F**).

The results further suggest that the expression levels of *NnPAL*, *NnC4H*, and *Nn4CL*, which belong to the general phenylpropanoid pathway, as well as of *NnRAS* and *NnCYP*, are not significantly correlated with the rosmarinic acid content in the two plant organs (**Table 3**). This is not surprising, taking into account that the majority of analyzed genes are related to the general phenylpropanoid pathway and are thus involved in providing the precursors for the biosynthesis of various classes of phenolic compounds. Furthermore, the differences in the quantitative content of the major phenolic compounds between leaves and inflorescences are less prominent than those recorded for iridoids.

**Table 3:** Correlations (*R* ^2^) between quantitative metabolomics data (for 4 selected phenolic compounds) and qPCR expression data for 7 rosmarinic acid biosynthesis-related genes in mature leaves and inflorescences of *Nepeta nuda* (*n*= 108).

Genes	Metabolites			
	Caffeic acid	Rosmarinic acid	Luteolin	Apigenin
***NnPAL***	0.70436	0.37304	0.41609	0.6189
***NnC4H***	0.44942	0.88650	0.44946	0.77645
***Nn4CL***	0.80174	0.81177	0.78283	0.89404
***NnTAT***	0.03716	0.00140	0.04487	0.01887
***NnHPPR***	0.47873	0.22249	0.27942	0.42757
***NnRAS***	0.62464	0.74598	0.67462	0.56519
***NnCYP***	0.72381	0.83617	0.87822	0.66551

No significant correlations were observed.

*R* ^2^, coefficient of determination; *n*, total number of comparisons.

### 3.8 Possible ecological implications of differential phenolics’ composition in *N*. *nuda* inflorescences and leaves

The involvement of phenolic compounds in plants ranges from growth and development (e.g., cell wall thickening, hormone production, and pigmentation), reproduction (e.g., pigmentation, fruit flavoring, and fruit protection), defense against stressors (e.g., osmoregulation, UV protection, anti-herbivory roles, and antimicrobial activity) (Dixon, 2001; Singh et al., 2021), and leaf and petal senescence (Tyagi et al., 2020). The content of phenolics in plants is determined by various endogenous and environmental factors. Thus, high constitutive and inducible leaf flavonoid content has been correlated with insect and pathogen resistance (Treutter, 2005). The function of phenolics in flowers is particularly versatile: from antioxidant activity, through the protection from UV radiation, to providing diverse colors. Phenolics accumulated in flowers primarily protect from nectar robbers and enhance memory and foraging efficiency of pollinators or, as in the case of flavonoids, exhibit high antioxidant activities supporting pollinator well-being (Slavković and Bendahmane, 2023). Moreover, flavonoids can regulate biotic interactions with mutualists and antagonists and thus reduce herbivore attacks and infection (Cushnie and Lamb, 2005; Palmer-Young et al., 2019).

Rosmarinic acid, which is by far the most abundant compound in *N. nuda*, is known for its antioxidant (Erkan et al., 2008; Muñoz-Muñoz et al., 2013), antimicrobial (Bais et al., 2002; Ivanov et al., 2022), insecticidal activities (Khan et al., 2019; Darrag et al., 2022), and various health promoting effects (Amoah et al., 2016; Alagawany et al., 2017; Nadeem et al., 2019). Rosmarinic acid displays insecticidal effects against the pea aphid, *Acyrthosiphon pisum* (Khan et al., 2019), and is a highly potent feeding deterrent for the tobacco hornworm (*Manduca sexta*) (Simmonds et al., 2019). Salvigenin, nepetoidin B, and rosmarinic acid display respectable insecticidal activities against the red palm weevil (*Rhynchophorus ferrugineus*) (Darrag et al., 2022). Conversely, this compound has been reported to reduce honey bee mortality caused by pesticides (Ulziibayar et al., 2022).

## 4 Conclusions

Inflorescences and leaves of *Nepeta nuda* showed a differential investment in the constitutive production of phenolics and terpenes, based on their chemical profiles, which emphasizes the importance of the chemical profiling of different plant organs and encompassing both groups of metabolites to properly characterize the species’ metabolome. The data strongly indicate that plants prioritize constitutive production of floral metabolites, whose qualitative and quantitative content is, to a large extent, conserved among different populations. *NnG8H* and *NnNEPS1-3*, are recognized as important regulatory points responsible for the increased content of iridoids in inflorescences.

The presented results lead to the unambiguous conclusion that specialized metabolism in leaves is more reprogrammable in response to differential growth conditions than that in inflorescences. Therefore, vegetative parts are highlighted as more convenient objects for studying the environmentally-driven chemical diversity among *N. nuda* populations. However, the specific mechanisms and the exact environmental cues associated with the organ-specific chemical composition in *N. nuda* remain largely unexplored. Due to its rich metabolite profile and a potential ornamental value, *N. nuda* can be a suitable alternative to *N. cataria*, which is the most intensively cultivated *Nepeta* species. The information about the intra-individual (organ-specific) chemical specificities of *N. nuda* provides strategic tools for growers to manipulate the phenology of this medicinal crop and achieve a higher productivity of metabolites of interest. Given the importance of iridoids as powerful insect repellents, future studies should be conducted towards optimization of agronomic practices to maximize yields in versatile geographic regions and to facilitate the integration of iridoid-containing biopesticides into pest management systems and other applications related to human health.

## Data Availability

The datasets presented in this study can be found in online repositories. The names of the repository/repositories and accession number(s) can be found in the article/**Supplementary Material**.
