# Supplementary material for: Molecular background of the diverse metabolic profiles in leaves and inflorescences of naked catmint (Nepeta nuda L.)
Source: Front Plant Sci. 2024 Nov 28;15:1452804. doi: 10.3389/fpls.2024.1452804 (PMC11634604; doi:10.3389/fpls.2024.1452804)
Supplement: Supplementary file 1 [file DataSheet1.docx]

Supplementary Material

# Supplementary Table 1. Accession numbers of sequences and primer combinations used for qPCR analysis of iridoid- and phenolic-biosynthetic genes in *N. nuda*.

| **Sequence abbreviation** | Accession number | Primer name | Primer sequence (5’-3’) | Target gene |
| --- | --- | --- | --- | --- |
| **NnGAPDH** | MN936120 | NnGAPDH F | GGCAAAGTTCTCCCTGCTCT | GAPDH |
|  |  | NnGAPDH R | ATACTCGACCTGTTGTCGCC |  |
| **NnGPPS** | PP935157 | NnGPPS F | CCGGTCAGTTTCTCGACCTC | GPPS |
|  |  | NnGPPS R | GGCTCGCCCATATTTCCTCA |  |
| **NnGES** | PP935158 | NnGES F | TGGTTCAATGGTGGAATTGC | GES |
|  |  | NnGES R | GAGATCATCCCAAAGGCGAA |  |
| **NnG8H** | OR818587 | NnG8H F | CGTCGCCAAAGAAGTCCTCCA | G8H |
|  |  | NnG8H R | CGGAGTACTTGAACTGGTCGT |  |
| **Nn8HGO** | PP935159 | Nn8HGO F | TGGTTAAGGTGGATCCGAAG | 8HGO |
|  |  | Nn8HGO R | CTTTTGCCTTATCGCGTCTC |  |
| **NnISY** | PP935160 | NnISY F | GCCCATGGAAGGTATACGGG | ISY |
|  |  | NnISY R | GATAGCTTCGCCTCCACCTC |  |
| **NnMLPL** | PP935161 | NnMLPL F | TCATTTCCCAAGGCTTTGCC | MLPL |
|  |  | NnMLPL R | CTCTCCTTCGTCCTCGAACC |  |
| **NnNEPS1** | PP935162 | NnNEPS1 F | CTCAAAGGGGTCACCCTCAC | NEPS1 |
|  |  | NnNEPS1 R | ATGTCCAGTGATGAACGCGG |  |
| **NnNEPS2** | PP935163 | NnNEPS2 F | ATGAGCCAATCTGAGTCCGC | NEPS2 |
|  |  | NnNEPS2 R | ATCAATGTATCCGCCCCTCG |  |
| **NnNEPS3** | PP935164 | NnNEPS3 F | GGCTAACAATTCAGTGATGATGAAG | NEPS3 |
|  |  | NnNEPS3 R | CCGTACCTCACGAACATGC |  |
| **NnPAL** | OR855708 | NnPAL F | GTGGAGGAGTTCAGGAAGCC | PAL |
|  |  | NnPAL R | TCGCTGCTGGCCTTGAC |  |
| **NnC4H** | OR855709 | NnC4H F | GCGCAGAGCTTCGAGTACAA | C4H |
|  |  | NnC4H R | GCTCACGAGCTTCTTCCTCT |  |
| **Nn4CL2** | OR855710 | Nn4CL2 F | AAGGATACCAAGTTGCTCCG | 4CL2 |
|  |  | Nn4CL2 R | TTCCAGATGGATTTTTGGGGA |  |
| **NnHPPR** | OR855711 | NnHPPR F | GGGGACCCCATGTTGATGAG | HPPR |
|  |  | NnHPPR R | CGGGTTCCCTTTCGAAGACA |  |
| **NnTAT** | OR855712 | NnTAT F | GGACTGCGCGACACAATATG | TAT |
|  |  | NnTAT R | CCCCATCCCGTTGCTTCATC |  |
| **NnRAS** | OR855713 | NnRAS F | CCTCGAGATCGACTGCAACA | RAS |
|  |  | NnRAS R | CCACCGCATTTGAAACGAGT |  |
| **NnCYP98A14** | OR855714 | NnCYP98A14 F | ACGAGGCTGGTTTTTGGGAA | CYP98A14 |
|  |  | NnCYP98A14 R | GGGTGTGCTCCTGCATGATT |  |

**Supplementary Table 2.** UHPLC-ESI-QToF-MS data on metabolites identified in metanol extracts of *Nepeta nuda* leaves and inflorescences.

| **No** | **Compound name** | ***t*_R_, min** | **Molecular formula, [M±H]^±^** | **Calculated mass, *m/z*** | **Exact mass, *m/z*** | **Δ mDa** | **MS^2^ fragments, (% base peak)** | **L** | **I** | **Previously reported in *Nepeta* sp.** |
| --- | --- | --- | --- | --- | --- | --- | --- | --- | --- | --- |
| ***Hydroxybenzoic acid derivatives*** | | | | | | | | | | |
| **1** | **Galloyl hexoside** | 1.41 | C_13_H_15_O_10_^–^ | 331.06655 | 331.07343 | –6.88 | 124.01732(17), 125.02673(94), **168.00944**(100), 169.01204(14), 313.05871(7) | ✚ | ✚ | Mouhajir et al., 2001 |
| **2** | **Gallic acid** | 1.47 | C_7_H_5_O_5_^–^ | 169.01370 | 169.02035 | –6.65 | 107.01403(14), **125.02441**(100) | ✚ | ✚ | Kaska et al., 2011 |
| **3** | **Dihydroxybenzoic acid** | 2.15 | C_7_H_5_O_4_^–^ | 153.01933 | 153.02445 | –5.12 | **108.02182**(100), 109.02969(84) | ✚ | **–** | Mišić et al., 2015 |
| **4** | **Dihydroxybenzoic acid hexoside** | 2.42 | C_13_H_15_O_9_^–^ | 315.07216 | 315.07717 | –5.01 | **108.02285**(100), 123.04534(9), 152.01279(66) | ✚ | ✚ | Aničić et al., 2021 |
| **5** | **Vanillic acid hexoside** | 3.23 | C_14_H_17_O_9_^–^ | 329.08781 | 329.09059 | –2.79 | **108.02225**(100), 123.04585(40), 152.01204(67), 167.03435(36) | ✚ | ✚ | Petrović et al., 2024 |
| **6** | **Hydroxybenzoic acid** | 3.77 | C_7_H_5_O_3_^–^ | 137.02390 | 137.02642 | –2.52 | NA | ✚ | ✚ | Mišić et al., 2015 |
| **7** | **Dihydroxybenzoic acid hexoside isomer** | 4.65 | C_13_H_15_O_9_^–^ | 315.07216 | 315.07757 | –5.42 | **109.03019**(100), 135.00806(5), 153.02023(29) | ✚ | ✚ | Mišić et al., 2015 |
| **8** | **Dihydroxybenzoic acid isomer** | 5.35 | C_7_H_5_O_4_^–^ | 153.01933 | 153.02154 | –2.21 | **109.02945**(100), 135.00889(22) | ✚ | ✚ | Mišić et al., 2015 |
| **9** | **Hydroxybenzoic acid hexoside** | 5.86 | C_13_H_15_O_8_^–^ | 299.07670 | 299.08413 | –7.43 | **137.02614**(100) | ✚ | ✚ | Aničić et al., 2021 |
| ***Hydroxycinnamic acid derivatives*** | | | | | | | | | | |
| **10** | **Syringic acid hexoside** | 2.45 | C_15_H_19_O_10_^–^ | 359.09837 | 359.10244 | –4.07 | 123.04584(29), 135.04582(42), 179.03633(84), **197.04727**(100) | ✚ | ✚ | Aničić et al., 2021 |
| **11** | **Caffeoyltartaric acid** | 3.77 | C_13_H_11_O_9_^–^ | 311.04030 | 311.05026 | –9.96 | **135.04587**(100), 149.00992(14), 179.03556(17) | ✚ | ✚ | Petrović et al., 2024 |
| **12** | **Caffeoyltartronic acid** | 4.31 | C_12_H_9_O_8_^–^ | 281.03029 | 281.03386 | –3.57 | **109.03139**(100), 133.03049(4), 135.04562(3), 149.06061(3), 161.02493(4) | ✚ | ✚ | Snook et al., 1993 |
| **13** | **Caffeic acid hexoside** | 4.71 | C_15_H_17_O_9_^–^ | 341.08739 | 341.09316 | –5.77 | 133.03004(22), 135.04525(13), **161.02543**(100), 179.03550(8) | **–** | ✚ | Aničić et al., 2021 |
| **14** | **Caffeoylglycolic acid** | 4.84 | C_11_H_9_O_6_^–^ | 237.04046 | 237.04355 | –3.09 | **133.03031**(100), 149.06131(6), 161.02503(51) | ✚ | ✚ | Snook et al., 1993 |
| **15** | **Umbelliferone** | 5.45 | C_9_H_7_O_3_^+^ | 163.03897 | 163.04213 | –3.16 | 103.05416(4), 107.04999(20), **117.03475**(100), 135.04366(28) | ✚ | ✚ | Dienaite et al., 2018 |
| **16** | **Caffeoyltartronic acid isomer** | 5.46 | C_12_H_9_O_8_^–^ | 281.03029 | 281.03442 | –4.13 | **109.02964**(100), 135.04576(3), 149.06090(3) | ✚ | ✚ | Snook et al., 1993 |
| **17** | **Sinapic acid** | 6.00 | C_11_H_11_O_5_^–^ | 223.06120 | 223.06334 | –2.14 | 119.99436(15), 121.04802(18), **135.04597**(100), 145.05773(16), 151.01879(20) | ✚ | ✚ | Aničić et al., 2021 |
| **18** | **Caffeic acid hexoside isomer** | 6.13 | C_15_H_17_O_9_^–^ | 341.08739 | 341.09375 | –6.36 | **135.04617**(100), 149.06111(4), 161.02557(22), 177.05660(10), 179.03657(73) | ✚ | ✚ | Aničić et al., 2021 |
| **19** | **Dihydrocaffeic acid hexoside** | 6.20 | C_15_H_19_O_9_^–^ | 343.10294 | 343.10824 | –5.30 | 119.05022(6), 137.06134(68), **181.05186**(100) | ✚ | ✚ | Fraga et al., 2017 |
| **20** | **Caffeic acid** | 6.33 | C_9_H_7_O_4_^–^ | 179.03440 | 179.03911 | –4.71 | 107.05033(9), 117.03491(7), **135.04619**(100) | ✚ | ✚ | Aničić et al., 2021 |
| **21** | **Feruloyltartaric acid** | 6.39 | C_14_H_13_O_9_^–^ | 325.05606 | 325.06166 | –5.60 | 119.05055(58), **134.03769**(100), 149.02713(7), 163.04084(14), 178.02764(4), 193.05174(13) | ✚ | ✚ | Zengin et al., 2021 |
| **22** | **Ethyl caffeate** | 6.90 | C_11_H_11_O_4_^–^ | 207.06628 | 207.06817 | –1.88 | **135.04752**(100), 163.04087(8) | ✚ | ✚ | Fraga et al., 2017 |
| **23** | **Caffeoylmalic acid** | 6.95 | C_13_H_11_O_8_^–^ | 295.04540 | 295.05022 | –4.82 | **135.04607**(100), 163.04099(12) | ✚ | ✚ | Petrović et al., 2024 |
| **24** | **Rosmarinic acid hexoside** | 7.74 | C_24_H_25_O_13_^–^ | 521.12952 | 521.13701 | –7.49 | 135.04608(93), 161.02531(99), **179.03650**(100), 197.04739(41), 341.08715(8), 359.08803(21) | **–** | ✚ | NA |
| **25** | **Feruloylmalic acid** | 7.89 | C_14_H_13_O_8_^–^ | 309.06100 | 309.06740 | –6.40 | 117.03496(12), **134.03810**(100), 147.03008(7), 178.02696(6), 193.05085(8) | ✚ | ✚ | Petrović et al., 2024 |
| **26** | **Rosmarinic acid** | 8.56 | C_18_H_15_O_8_^–^ | 359.07671 | 359.08532 | –8.61 | 123.04620(13), 133.03059(17), 135.04630(27), **161.02576**(100), 179.03672(26), 197.04684(13) | ✚ | ✚ | Aničić et al., 2021 |
| **27** | **Ferulic acid** | 8.63 | C_10_H_9_O_4_^–^ | 193.05063 | 193.05230 | –1.66 | **133.03042**(100), 161.02513(14) | **–** | ✚ | Alimpić Aradski et al., 2023 |
| **28** | **Nepetoidin A or B** | 10.31 | C_17_H_13_O_6_^–^ | 313.07124 | 313.07606 | –4.82 | 123.04611(10), 133.03063(55), 151.04076(12), **161.02620**(100) | **–** | ✚ | Aničić et al., 2021 |
| ***Iridoid glycosides*** | | | | | | | | | | |
| **29** | **Aucubin** | 5.73 | C_15_H_21_O_9_^–^ | 345.11911 | 345.12296 | –3.85 | 101.02488(58), 113.02496(49), **119.04784**(100), 137.06027(11), 183.06797(3) | ✚ | ✚ | El-Moaty et al., 2010 |
| **30** | **6α-Hydroxyadoxoside** | 5.79 | C_17_H_25_O_11_^–^ | 405.14024 | 405.14119 | –0.95 | 153.05597(6), 175.02649(5), 179.07328(7), **197.08320**(100) | ✚ | ✚ | Goldansaz et al., 2019 |
| **31** | **Nepetanudoside C + HCOOH** | 6.13 | C_17_H_23_O_10_^–^ | 387.12967 | 387.12974 | –0.07 | 101.02413(94), 113.02500(94), 146.03664(60), **161.05728**(100), 165.05628(21), 179.07016(50) | ✚ | ✚ | Takeda et al., 1995 |
| **32** | **5-Deoxylamiol** | 6.47 | C_16_H_25_O_9_^–^ | 361.15041 | 361.15130 | –0.89 | 101.02516(19), 115.04080(10), 119.08233(16), **137.09782**(100), 181.08803(11), 199.09816(18) | ✚ | ✚ | Petrović et al., 2024 |
| **33** | **1,5,9-*epi*-Deoxyloganic acid hexoside + HCOOH** | 6.61 | C_23_H_35_O_16_^–^ | 567.19257 | 567.19962 | –7.05 | 109.06523(5), 135.08222(15), 153.09337(54), 197.08377(90), 239.09320(5), **359.13759**(100) | ✚ | ✚ | Takeda et al., 1998 |
| **34** | **Nepetanudoside А + HCOOH** | 7.01 | C_18_H_27_O_12_^–^ | 435.15080 | 435.15845 | –7.65 | **101.02477**(100), 183.06744(5), 227.09479(25) | ✚ | ✚ | Takeda et al., 1995 |
| **35** | **1,5,9-*epi*-Deoxyloganic acid** | 7.41 | C_16_H_23_O_9_^–^ | 359.13476 | 359.13819 | –3.43 | 109.06682(9), **135.08297**(100), 153.09284(26), 197.08194(5) | ✚ | ✚ | Aničić et al., 2021 |
| **36** | **Methyl-1,5,9-*epi*-Deoxyloganic acid** | 7.54 | C_17_H_27_O_9_^+^ | 375.16496 | 375.16420 | 0.76 | 135.08002(60), 137.09618(31), 153.09043(26), 163.07536(90), **181.08540**(100), 199.09127(13) | ✚ | **–** | Nagy et al., 1998 |
| **37** | **Nepetaside** | 7.74 | C_16_H_25_O_8_^–^ | 345.15549 | 345.15841 | –2.92 | **101.02493**(100), 113.02503(75), 119.03541(32), 167.10828(32), 185.11870(51) | ✚ | ✚ | Xie et al., 1988 |
| **38** | **6-Deoxylamioside** | 7.82 | C_18_H_27_O_10_^–^ | 403.16097 | 403.16099 | –0.01 | 179.10829(93), **197.11859**(100), 223.09835(52), 241.10981(27) | ✚ | ✚ | Aničić et al., 2021 |
| **39** | **Caffeoyl-1,5,9-*epi*-deoxyloganic acid** | 8.76 | C_25_H_29_O_12_^–^ | 521.16593 | 521.17181 | –5.88 | 135.07677(11), 153.09214(10), **161.02525**(100), 179.0377(9), 197.07641(7), 323.07601(7) | **–** | ✚ | NA |
| ***Iridoid aglycones*** | | | | | | | | | | |
| **40** | **5,9-Dehydronepetalactone** | 6.46 | C_10_H_13_O_2_^+^ | 165.09101 | 165.09134 | –0.33 | 103.05508(79), **105.06905**(100), 107.07625(61), 109.07648(13), 119.09073(70), 122.06183(18) | ✚ | ✚ | Srivastava et al., 2021 |
| **41** | **Nepetaside aglycone acetate** | 6.87 | C_12_H_19_O_4_^+^ | 227.12830 | 227.13202 | –3.72 | 105.07057(80), **107.08021**(100), 121.08498(48), 131.08589(93), 145.10006(26), 149.09439(53) | ✚ | ✚ | Xie et al., 1988 |
| **42** | **Loganetin** | 7.01 | C_11_H_17_O_5_^+^ | 229.10705 | 229.11138 | –4.33 | **105.07101**(100), 115.05545(16), 133.06566(50), 161.06027(14) | ✚ | ✚ | Petrović et al., 2024 |
| **43** | **Deoxygeniposide aglycone** | 7.01 | C_11_H_15_O_4_^+^ | 211.09700 | 211.10148 | –4.48 | 103.05506(23), **105.07104**(100), 115.05543(13), 133.06522(33), 135.08059(25), 161.06026(11) | ✚ | ✚ | Takeda et al., 1995 |
| **44** | **7-Deoxyloganetic acid** | 7.34 | C_10_H_15_O_4_^+^ | 199.09649 | 199.09970 | –3.21 | 105.07036(75), 107.08064(88), 111.07725(25), **115.05532**(100), 135.08059(58), 163.07571(22) | ✚ | ✚ | Murai et al., 1984 |
| **45** | **Nepetaracemoside B aglycone** | 7.35 | C_10_H_13_O_3_^+^ | 181.08650 | 181.09269 | –6.19 | **105.06972**(100), 107.07002(44), 115.05538(67), 123.07979(61), 125.04424(16), 151.03903(63) | ✚ | ✚ | Takeda et al., 1999 |
| **46** | **De-4-methylnepetalactol** | 8.76 | C_9_H_15_O_2_^+^ | 155.10666 | 155.10792 | –1.26 | 107.08620(11), **109.10239**(100) | ✚ | ✚ | Waller and Johnson, 1984 |
| **47** | **7-Deoxyloganetin** | 9.06 | C_11_H_15_O_4_^–^ | 211.09700 | 211.10073 | –3.73 | **101.02417**(100), 107.03404(13), 109.07041(16), 121.07518(8), 123.06118(15) | **–** | ✚ | Murai et al., 1984 |
| **48** | **Nepetalic acid** | 9.61 | C_10_H_15_O_3_^–^ | 183.10267 | 183.10321 | –0.54 | 107.05100(92), 121.06682(42), 135.08171(27), **137.09772**(100), 165.09377(41) | ✚ | ✚ | Patel et al., 2022 |
| **49** | ***trans*,*cis*-Nepetalactone** | 9.70 | C_10_H_15_O_2_^+^ | 167.10666 | 167.10866 | –2.01 | 103.05507(43), **105.07091**(100), 107.07640(13), 119.08617(19), 121.10107(22) | ✚ | ✚ | Alimpić Aradski et al., 2023 |
| **50** | **Nepetonic acid** | 10.10 | C_9_H_13_O_3_^–^ | 169.08702 | 169.08904 | –2.02 | 123.08205(15), **125.09862**(100), 151.07836(21) | ✚ | ✚ | Goldansaz et al., 2019 |
| **51** | **Dihydronepetalactone** | 10.92 | C_10_H_17_O_2_^+^ | 169.12231 | 169.12267 | –0.36 | 105.07085(11), 107.08429(8), 109.10045(30), 121.10647(8), **123.11750**(100) | **–** | ✚ | Regnier et al., 1967 |
| **52** | ***cis*,*trans*-Nepetalactone** | 11.18 | C_10_H_15_O_2_^+^ | 167.10666 | 167.10818 | –1.52 | 105.07099(10), **111.04573**(100), 121.09798(3) | ✚ | **–** | Alimpić Aradski et al., 2023 |
| **53** | **Nepetalactol acetate** | 11.19 | C_12_H_19_O_3_^+^ | 211.13287 | 211.13652 | –3.65 | **105.07029**(100), 107.08509(47), 119.08591(43), 125.03622(16), 128.03647(18), 133.08077(45) | ✚ | **–** | Salehi et al., 2007 |
| **54** | **Methyl acetal of nepetalic acid** | 11.99 | C_11_H_19_O_3_^+^ | 199.13287 | 199.13348 | –0.61 | **105.07083**(100), 109.10164(54), 121.10184(96), 123.11649(11), 125.05883(9), 139.11142(8) | **–** | ✚ | Barhoumi et al., 2023 |
| **55** | ***trans*,*cis*-Nepetalactone** | 12.07 | C_10_H_15_O_2_^+^ | 167.10666 | 167.10855 | –1.89 | 105.07139(15), **111.04675**(100), 121.09868(5) | ✚ | ✚ | Alimpić Aradski et al., 2023 |
| ***Flavonoid glycosides*** | | | | | | | | | | |
| **56** | **Luteolin 7-*O*-(6"-hexuronyl)-hexoside** | 5.59 | C_27_H_27_O_17_^–^ | 623.12486 | 623.13153 | –6.68 | **109.03032**(100), 149.06083(4), 161.02436(11), 179.03545(3), 193.05110(20) | ✚ | ✚ | Petrović et al., 2024 |
| **57** | **Luteolin 7-*O*-(2"-hexuronyl)-hexuronide** | 7.21 | C_27_H_25_O_18_^–^ | 637.10412 | 637.11163 | –7.51 | 113.02472(21), 175.02568(8), 193.03640(21), **285.04251**(100), 351.05935(77) | ✚ | **–** | Dienaite et al., 2018 |
| **58** | **Luteolin 7-*O*-hexuronide** | 7.88 | C_21_H_17_O_12_^–^ | 461.07200 | 461.07835 | –6.35 | **285.04335**(100) | **–** | ✚ | Tomas-Barberan et al., 1992 |
| **59** | **Luteolin 7-*O*-(2"-caffeoyl)-hexuronide** | 9.26 | C_30_H_23_O_15_^–^ | 623.10424 | 623.11285 | –8.61 | **161.02524**(100), 179.03660(6), 285.04211(61), 337.05827(36), 443.06410(18) | **–** | ✚ | NA |
| **60** | **Apigenin 7-*O*-(2"-acetyl)-hexuronide** | 9.57 | C_23_H_21_O_12_^+^ | 489.10333 | 489.11066 | –7.33 | 113.02405(3), 159.02944(4), **271.06278**(100) | **–** | ✚ | NA |
| ***Flavonoid aglycones*** | | | | | | | | | | |
| **61** | **Luteolin** | 9.39 | C_15_H_9_O_6_^–^ | 285.03990 | 285.04702 | –7.12 | 107.01498(18), **133.03108**(100), 149.02488(14), 151.00436(38), 175.04083(18), 199.04055(13) | **–** | ✚ | Ahmed et al., 2006 |
| **62** | **Thymusin** | 10.38 | C_17_H_13_O_7_^–^ | 329.06615 | 329.07465 | –8.50 | 117.03536(7), 151.00456(4), 179.00091(27), 271.0266(33), **299.02439**(100), 314.04460(7) | ✚ | ✚ | Aničić et al., 2021 |
| **63** | **Cirsimaritin** | 11.19 | C_17_H_13_O_6_^–^ | 313.07124 | 313.07807 | –6.83 | 163.00548(13), 255.03267(19), 269.04769(6), **283.02700**(100), 297.04336(15), 298.04845(8) | ✚ | ✚ | Aničić et al., 2021 |
| **64** | **Xanthomicrol** | 11.39 | C_18_H_17_O_7_^+^ | 345.09745 | 345.10480 | –7.35 | 148.05295(7), 269.04548(9), **284.07100**(100), 312.06655(81), 315.05252(7), 330.07592(48) | **–** | ✚ | Aničić et al., 2021 |
| **65** | **Salvigenin** | 13.14 | C_18_H_17_O_6_^+^ | 329.10250 | 329.10925 | –6.75 | 133.06707(13), 240.07974(10), **268.07475**(100), 296.06940(78), 314.07896(22), 329.10246(24) | ✚ | ✚ | Tomas-Barberan et al., 1992 |
| ***Other metabolites*** | | | | | | | | | | |
| **66** | **Quinic acid** | 0.80 | C_6_H_7_O_7_^–^ | 191.01920 | 191.02455 | –5.35 | **111.00978**(100) | ✚ | ✚ | Aničić et al., 2021 |
| **67** | **3-(3,4-Dihydroxyphenyl)-lactic acid** | 1.98 | C_9_H_9_O_5_^–^ | 197.04555 | 197.04603 | –0.48 | 107.04994(12), 109.03039(17), 117.03462(7), 123.04566(93), **135.04557**(100) | ✚ | ✚ | Hou et al., 2002 |
| **68** | **Hexenyl-pentosyl-hexoside** | 2.09 | C_17_H_29_O_10_^–^ | 393.17662 | 393.18045 | –3.83 | 101.02318(23), 113.02680(26), 119.01591(12), 123.02787(3), **131.03518**(100), 161.04879(5) | ✚ | ✚ | Goldansaz et al., 2019 |
| **69** | **Schizonepetin** | 6.46 | C_10_H_15_O_3_^+^ | 183.10157 | 183.10308 | –1.51 | 105.07057(27), 107.08372(24), **109.10040**(100), 117.07044(13), 119.08775(34), 123.07086(10) | ✚ | ✚ | Ze et al., 2011 |
| **70** | **12-*O*-Hexosyl-jasmonate** | 6.87 | C_18_H_27_O_9_^–^ | 387.16606 | 387.16637 | –0.32 | **101.02502**(100), 113.02505(72), 119.03558(42), 163.11382(51), 207.10349(81) | ✚ | ✚ | Aničić et al., 2021 |
| **71** | **Schizonepetin isomer** | 7.48 | C_10_H_15_O_3_^+^ | 183.10157 | 183.10547 | –3.90 | 105.07086(100), 107.07648(92), 109.07756(52), 117.07182(45), 119.08400(48), 121.09486(54) | ✚ | ✚ | Ze et al., 2011 |
| **72** | **2,5-Dimethyl-hexanedioic acid** | 7.49 | C_8_H_13_O_4_^–^ | 173.08193 | 173.08272 | –0.79 | **109.06619**(100), 111.08276(73), 127.05401(4), 129.09233(6) | ✚ | ✚ | Stepaneco et al., 1980 |
| **73** | **2-Carboxy-3-methyl-cyclopentaneacetic acid** | 8.15 | C_9_H_13_O_4_^–^ | 185.08140 | 185.08673 | –5.33 | 117.02066(13), 121.04882(13), 123.06020(68), 123.08541(57), **141.09308**(100), 167.04390(13) | ✚ | ✚ | Tagawa and Murai, 1983 |
| **74** | **Argolic acid A rhamnoside** | 8.76 | C_16_H_27_O_8_^–^ | 347.17114 | 347.17451 | –3.37 | 101.02454(9), **139.11356**(100), 163.06222(17), 183.10372(88), 201.11441(82) | ✚ | ✚ | Petrović et al., 2024 |
| **75** | **2-Carboxy-α,3-dimethyl-cyclopentaneacetic acid** | 9.03 | C_10_H_15_O_4_^–^ | 199.09700 | 199.10188 | –4.88 | 137.09790(29), **155.10907**(100) | ✚ | ✚ | Eisenbraun et al., 1988 |
| **76** | **9-Oxononanoic acid** | 9.12 | C_9_H_15_O_3_^–^ | 171.10267 | 171.10333 | –0.67 | 123.08108(6), **125.09811**(100) | ✚ | ✚ | Kilic et al., 2011 |
| **77** | **2-Carboxy-3-methyl-cyclopentaneacetic acid isomer** | 9.23 | C_9_H_13_O_4_^–^ | 185.08140 | 185.08669 | –5.29 | 125.09854(33), **141.09333**(100) | ✚ | ✚ | Tagawa and Murai, 1983 |
| **78** | **Argolic acid A** | 9.30 | C_10_H_17_O_4_^–^ | 201.11323 | 201.11437 | –1.14 | 111.07898(26), 123.05859(5), 137.10383(28), **139.11271**(100), 183.10100(35) | ✚ | ✚ | Ahmed et al., 2006 |
| **79** | **Argolic acid A methyl ether rhamnoside** | 9.37 | C_17_H_29_O_8_^–^ | 361.18679 | 361.19035 | –3.56 | 153.12884(41), 163.06281(16), **197.11928**(100), 215.13001(52) | ✚ | ✚ | Petrović et al., 2024 |
| **80** | **2-Carboxy-3-methyl-cyclopentaneacetic acid dimethyl ester** | 9.43 | C_11_H_19_O_4_^+^ | 215.12830 | 215.13305 | –4.75 | **109.10162**(100), 119.08582(30), 131.08523(9), 133.09821(6), 134.09641(6), 137.10016(7) | **–** | ✚ | Tagawa and Murai, 1983 |
| **81** | **Nepetalactam** | 10.07 | C_10_H_16_NO^+^ | 166.12264 | 166.12873 | –6.09 | **110.06329**(100), 150.06576(9) | ✚ | ✚ | Handjieva et al., 1996 |
| **82** | **2-Carboxy-α,3-dimethyl-cyclopentaneacetic acid dimethyl ester** | 10.45 | C_12_H_19_O_4_^–^ | 227.12888 | 227.12899 | –0.11 | 111.06175(16), 165.12273(11), **183.14644**(100) | ✚ | ✚ | Regnier et al., 1967 |

***t*_R_** – retention time (min); **Δ mDa** – mean mass accuracy; **NA** – not available; **L** – leaves; **I** – inflorescences. The **[M±H]^±^** column indicates in which ionization mode the corresponding compound was identified; in the **MS^2^ fragments** column, a dominant fragment is represented boldfaced.

## Supplementary Figure 1





**Supplementary Figure 1.** The proposed MS^2^fragmentation pattern of luteolin 7-*O*-(2"-caffeoyl)-hexuronide, displaying pseudomolecular ion [M−H]^−^ at of 623 *m/z*. Ions detected at 443 and 337 *m/z* are formed by the neutral loss of caffeic acid (−180 Da) and luteolin (−286 Da), respectively.

# References

Ahmed, A.A., Hassan, H.E., Hegazy, M.F., Tzakou, O., Couladis, M., Mohamed, A.E.-H.H., Abdella, M.A., Pare, P. (2006). Argolic acid A and argolic methyl ester B, two new cyclopentano-monoterpenes diol from *Nepeta argolica*. Nat Prod Com. 1, 523–526. DOI: 10.1177/1934578X0600100701.

Alimpić Aradski, A., Oalđe Pavlović, M., Janošević, D., Todorović, S., Gašić, U., Mišić, D., Pljevljakušić, D., Šavikin, K., Marin, P.D., Giweli, A., Duletić‐Laušević, S. (2023). Leaves micromorphology, chemical profile, and bioactivity of in vitro‐propagated *Nepeta cyrenaica* (Lamiaceae). Phytochem Anal. 34(6), 661-679. DOI: 10.1002/pca.3257.

Aničić, N., Gašić, U., Lu, F., Ćirić, A., Ivanov, M., Jevtić, B., Dimitrijević, M., Anđelković, B., Skorić, M., Nestorović Živković, J., Mao, Y., Liu, J., Tang, C., Soković, M., Ye, Y., Mišić, D. (2021). Antimicrobial and Immunomodulating Activities of Two Endemic *Nepeta* Species and Their Major Iridoids Isolated from Natural Sources. Pharmaceuticals. 14(5), 414. DOI: 10.3390/ph14050414.

Barhoumi, L. M., Al-Jaber, H. I., Zarga, M. H. A., Al-Qudah, M. A. (2023). A study of the chemical constituents of *Nepeta curviflora* from Jordan. Arab. J. Chem. 16(4), 104534. DOI:10.1016/j.arabjc.2022.104534.

Dienaitė, L., Pukalskienė, M., Matias, A.A., Pereira, C.V., Pukalskas, A., Venskutonis, P.R. (2018). Valorization of six *Nepeta* species by assessing the antioxidant potential, phytochemical composition and bioactivity of their extracts in cell cultures. J. Func. Foods. 45, 12-522.

Eisenbraun, E. J., Sullins, D. W., Browne, C. E., Shoolery, J. N. (1988). (4aS, 7S, 7aR)-Nepetalactam and (4aS, 7S, 7aR)-2-[(3R, 4R, 4aR, 7S, 7aR)-octahydro-4, 7-dimethyl-1-oxocyclopenta [c] pyran-3-yl] nepetalactam. Nitrogen analogs of nepetalactone and nepetalic. psi.-anhydride. J. Org. Chem. 53(17), 3968-3972. DOI: 10.1021/jo00252a016.

El-Moaty, H.I.A. (2010). Essential oil and iridoid glycosides of *Nepeta septemcrenata* Erenb. Journal of Natural Products, 3, 103–111. https://www.scinapse.io/papers/2188484169

Fraga, B.M., González-Coloma, A., Alegre-Gómez, S., López-Rodríguez, M., Amador, L.J., Díaz, C.E. (2017). Bioactive constituents from transformed root cultures of *Nepeta teydea*. Phytochem. 133, 59-68. DOI: 10.1016/j.phytochem.2016.10.008.

Goldansaz, S.M., Festa, C., Pagano, E., De Marino, S., Finamore, C., Parisi, O.A., Borrelli, F., Sonboli, A., D’Auria, M.V. (2019). Phytochemical and biological studies of *Nepeta asterotricha* Rech. f. (Lamiaceae): Isolation of nepetamoside. Molecules. 24(9), p.1684. DOI: 10.3390/molecules24091684.

Handjieva, N.V., Popov, S.S., Evstatieva, L.N. (1996). Constituents of Essential Oils from *Nepeta cataria* L., *N. grandiflora* MB and *N. nuda* L. J. Essent. Oil Res. 8, 639–643. DOI: 10.1080/10412905.1996.9701032.

Hou, Z.F., Tu, Y.Q, Li, Y. (2002). Three new phenolic compounds from *Nepeta prattii*. J. Chin. Chem. Soc. 49(2), 255-258. DOI: 10.1002/jccs.200200039.

Kaska, A., Deniz, N., Çiçek, M., Mammadov, R. (2018). Evaluation of antioxidant properties, phenolic compounds, anthelmintic, and cytotoxic activities of various extracts isolated from *Nepeta cadmea*: an endemic plant for Turkey. J. Food Sci. 83(6), 1552-1559. DOI: 10.1111/1750-3841.14167.

Kilic, O., Hayta, S., Bagci, E. (2011). Chemical composition of essential oil of *Nepeta nuda* L. subsp. nuda (Lamiaceae) from Turkey. Asian Journal of Chemistry. 23(6), 2788.

Mišić, D., Siler, B., Gašić, U., Avramov, S., Zivković, S., Nestorović Živković, J., Milutinović, M., Tešić, Z. (2015). Simultaneous UHPLC/DAD/(+/-)HESI-MS/MS analysis of phenolic acids and nepetalactones in methanol extracts of Nepeta species: a possible application in chemotaxonomic studies. Phytochem Anal. 26(1), 72-85. DOI: 10.1002/pca.2538.

Mouhajir, F., Pedersen, J. A., Rejdali, M., Towers, G. H. N. (2001). Phenolics in Moroccan Medicinal Plant Species as Studied by Electron Spin Resonance Spectroscopy. Pharm. Biol. 39:5, 391-398, DOI: 10.1076/phbi.39.5.391.5893

Murai, F., Tagawa, M., Damtoft, S., Jensen, S.R., Nielsen, B.J. (1984). (1R, 5R, 8S, 9S)-Deoxyloganic acid from *Nepeta cataria*. Chem. Pharm. Bull. 32, 2809–2814. DOI: 10.1248/cpb.32.2809.

Nagy, T., Kocsis, Á., Morvai, M., Szabó, L., Podányi, B., Gergely, A., Jerkovich, G. (1998). 2′-, 4′-, and 6′-O-substituted 1, 5, 9-epideoxyloganic acids from Nepeta grandiflora. Phytochemistry. 47(6), 1067-1072. DOI: 10.1016/S0031-9422(98)80074-5.

Patel, H., Gomes, E. N., Yuan, B., Lyu, W., Wu, Q., Simon, J. E. (2022). Investigation of volatile iridoid terpenes in *Nepeta cataria* L. (Catnip) Genotypes. Molecules. 27(20), 7057. DOI: 10.3390/molecules27207057.

Petrović, L., Skorić, M., Šiler, B., Banjanac, T., Gašić, U., Matekalo, D., Lukić, T., Nestorović Živković, J., Dmitrović, S., Aničić, N., et al. (2024). Patterns of Genetic Variation of *Nepeta nuda* L. from the Central Balkans: Understanding Drivers of Chemical Diversity. Plants. 13, 1483. https://doi.org/10.3390/plants13111483

Regnier, F. E., Waller, G. R., Eisenbraun, E. J. (1967). Studies on the composition of the essential oils of three *Nepeta* species. Phytochemistry. 6(9), 1281-1289. DOI: 10.1016/S0031-9422(00)86089-6.

Salehi, P., Sonboli, A, Allahyari, L. (2007). Antibacterial and antioxidant properties of the essential oil and various extracts of *Nepeta ispahanica* from Iran. J. Essent. Oil-Bear. Plants. 10(4), 324-331. DOI: 10.1080/0972060X.2007.10643563.

Snook, M.E., Blum, M.S., Whitman, D.W., Arrendale, R.F., Costello, C.E., Harwood, J.S. (1993). Caffeoyltartronic acid from catnip (*Nepeta cataria*): A precursor for catechol in lubber grasshopper (Romalea guttata) defensive secretions. Journal of chemical ecology. 19(9), 1957–1966. DOI: 10.1007/BF00983799

Srivastava, A., Gupta, S., Singh, S., Verma, R.S., Srivastava, R.K., Gupta, A.K., Lal, R.K. (2021). Genetic variability and elite line selection for high essential oil and nepetalactone content in catmint (*Nepeta cataria* L.). Am. J. Plant Sci. 12(7), 135-1154. DOI: 10.1016/S0031-9422(98)80074-5.

Stepanenko, G.A., Gusakova, S.D, Umarov, A.U. (1980). Composition of the coats and kernels of the seeds of *Nepeta pannonica* and *Lavandula vera*. Chem. Nat. Compd. 16, 434-439. DOI: 10.1007/BF00571031.

Takeda, Y., Morimoto, Y., Matsumoto, T., Honda, G., Tabata, M., Fujita, T., Otsuka, H., Sezik, E., Yesilada, E. (1995). Nepetanudoside, an iridoid glucoside with an unusual stereostructure from *Nepeta nuda* ssp. Albiflora. J. Nat. Prod. 58(8): 1217–1221. DOI: 10.1021/np50122a009.

Takeda, Y., Ooiso, Y., Masuda, T., Honda, G., Otsuka, H., Sezik, E., Yesilada, E. (1998). Iridoid and eugenol glycosides from *Nepeta cadmea*. Phytochem. 49, 787–791.DOI: 10.1016/S0031-9422(98)00125-3.

Takeda, Y., Kiba, Y., Masuda, T., Otsuka, H., Honda, G., Tagawa, M., Sezik, E., Yesilada, E. (1999). Nepetaracemosides A and B, iridoid glucosides from *Nepeta racemosa*. Chem. Pharm. Bull. 47(10), 1433-1435. DOI: 10.1248/cpb.32.2809.

Tagawa, M., Murai, F. (1983). 5-Epideoxyloganic acid from *Nepeta cataria*. Planta medica. 47(02), 109-111. DOI: 10.1055/s-2007-969965.

Tomas-Barberan, F.A., Gil, M.I., Ivancheva, S, Tomas-Lorente, F. (1992). External and vacuolar flavonoids from *Nepeta transcaucasica*. Biochem. Syst. Ecol. 20(6), 589-590. DOI: 10.1016/0305-1978(92)90013-4.

Waller, G.R., Johnson, R.D. (1984). Metabolism of nepetalactone and related compounds in *Nepeta cataria* L. and components of its bound essential oil. Proc Okla Acad Sci. 64, 49-56. https://ojs.library.okstate.edu/osu/index.php/OAS/article/view/5263/4932.

Xie, S., Uesato, S., Inouye, H., Fujita, T., Murai, F., Tagawa, M., Shingu, T. (1988). Absolute structure of nepetaside, a new iridoid glucoside from *Nepeta cataria*. Phytochem. 27(2), 469-472. DOI: 10.1016/S0031-9422(98)80074-5.

Ze, R., Tong, Z., Zhang, Z., Liu, Z., Xue, P., Xie, Y. (2011). Chemical constituents in essential oil of *Nepeta angustifolia*. Shizhen Guoyi Guoyao (Lishizhen Medicine and Materia Medica Research). 22(6), 1520–1521. DOI: 10.3969/j.issn.1008-0805.2011.06.114.

Zengin, G., Mahomoodally, M.F., Aktumsek, A., Jekő, J., Cziáky, Z., Rodrigues, M.J., Custodio, L., Polat, R., Cakilcioglu, U., Ayna, A., Gallo, M. (2021). Chemical profiling and biological evaluation of *Nepeta baytopii* extracts and essential oil: An endemic plant from Turkey. Plants. 10(6), 1176. DOI: 10.3390/plants10061176.
